# Different Gene Expressions of Resistant and Susceptible Hop Cultivars in Response to Infection with a Highly Aggressive Strain of *Verticillium albo-atrum*

**DOI:** 10.1007/s11105-014-0767-4

**Published:** 2014-08-17

**Authors:** Sara Cregeen, Sebastjan Radisek, Stanislav Mandelc, Boris Turk, Natasa Stajner, Jernej Jakse, Branka Javornik

**Affiliations:** 1 Agronomy Department, Biotechnical Faculty, University of Ljubljana, Jamnikarjeva 101, SI-1000 Ljubljana, Slovenia; 2 Slovenian Institute for Hop Research and Brewing, Cesta ŽalskegaTabora 2, SI-3320 Žalec, Slovenia

**Keywords:** Hops, Verticillium wilt, Differential gene expression, RT-qPCR, Colonization

## Abstract

Verticillium wilt has become a serious threat to hop production in Europe due to outbreaks of lethal wilt caused by a highly virulent strain of *Verticillium albo-atrum*. In order to enhance our understanding of resistance mechanisms, the fungal colonization patterns and interactions of resistant and susceptible hop cultivars infected with *V. albo-atrum* were analysed in time course experiments. Quantification of fungal DNA showed marked differences in spatial and temporal fungal colonization patterns in the two cultivars. Two differential display methods obtained 217 transcripts with altered expression, of which 84 showed similarity to plant proteins and 8 to fungal proteins. Gene ontology categorised them into cellular and metabolic processes, response to stimuli, biological regulation, biogenesis and localization. The expression patterns of 17 transcripts with possible implication in plant immunity were examined by real-time PCR (RT-qPCR). Our results showed strong expression of genes encoding pathogenesis-related (PR) proteins in susceptible plants and strong upregulation of genes implicated in ubiquitination and vesicle trafficking in the incompatible interaction and their downregulation in susceptible plants, suggesting the involvement of these processes in the hop resistance reaction. In the resistant cultivar, the RT-qPCR expression patterns of most genes showed their peak at 20 dpi and declined towards 30 dpi, comparable to the gene expression pattern of *in planta* detected fungal protein and coinciding with the highest fungal biomass in plants at 15 dpi. These expression patterns suggest that the defence response in the resistant cultivar is strong enough at 20 dpi to restrict further fungus colonization.

**Electronic supplementary material:**

The online version of this article (doi:10.1007/s11105-014-0767-4) contains supplementary material, which is available to authorized users.

## Introduction

*Verticillium albo-atrum* is a soil-borne pathogenic fungus that causes a vascular wilt disease in a broad range of plants, including economically important crops. The fungus infects plants by entering the xylem vessels through the roots after germination of the soil-resting structure. Colonization is enhanced by the production of spores spreading through the plants by the transpiration flow and by their subsequent germination. The fungus colonizes the plant’s entire vascular system, causing plant stunting and wilting, vascular browning and foliar chlorosis and necrosis (Pegg and Brady 2002; Fradin and Thomma 2006).

The pathogen can be a major limiting factor in the production of many crops, such as hops (*Humulus lupulus* L.), in which it causes mild (fluctuating) and lethal (progressive) forms of Verticillium wilt. Particularly high economic damage in European hop production has been caused by outbreaks of a highly virulent strain of *V. albo-atrum*, which induces lethal wilt symptoms, with rapid plant withering (Radisek et al. 2006). Host resistance is one of the most important means of disease management. Genetically based resistance to Verticillium wilt has been discovered in several crops (cotton, tomato, potato, sunflower, strawberry, oilseed rape, hop, lettuce) and has been bred into commercial varieties as a polygenic or monogenic trait (Wang et al. 2008; Schaible et al. 1951; Radi and Gulya 2007; Rygulla et al. 2008; Neve 1991; Hayes et al. 2011; Jakse et al. 2013). The best characterized is Ve-mediated resistance in tomato, in which cloned gene *Ve1* codes for a receptor-like protein and confers resistance to *Verticillium dahliae* and *V. albo-atrum* (Kawchuk et al. 2001; Fradin et al. 2009). In addition, an Ave1 effector was discovered in *V. dahliae,* which is recognized by the plant Ve1 receptor and triggers an immune response (de Jonge et al. 2012).

Colonization of susceptible and resistant plants is fully systemic and similar in terms of fungal biomass after infection, but resistant plants are later able to suppress fungal growth and spread. Plants respond to the invading pathogen with physical barriers, by producing depositions in cell walls and blockages of xylem vessels and by chemical defence, synthesising antimicrobial substances (Pegg and Brady 2002; Fradin and Thomma 2006).

These responses have been studied in various host-Verticillium pathosystems, by means of differential expression analysis. In tomato, transcriptomic analysis using a complementary DNA (cDNA) array of 8,500 genes was carried out on stem tissue of a susceptible tomato variety challenged by two different *V. dahliae* strains, causing susceptible and tolerant interactions. Induced pathogenesis related (PR) and other genes were identified, and some of them were shown to be strongly upregulated in susceptible plants, repressed in the tolerant interaction and unchanged in resistant tomato lines at 10 days post inoculation (dpi) (Robb et al. 2007; Robb et al. 2012). Transcription profiles by commercial tomato array containing over 22,000 transcripts were assessed in tomato-*V. dahliae* compatible and incompatible interactions (van Esse et al. 2009). Different responses were found in the two interactions, with a much higher number of differentially expressed genes in the compatible than in the resistant interaction. Identified induced genes were implicated in photorespiration, hypoxia, glycoxylate metabolism and auxin signalling. Measurements of an early resistance response of tomato to invading *V. dahlia* have shown the production of H_2_O_2_ with the peak at 2 h post inoculation (hpi) and induction of peroxidase (2 hpi), both probably involved in intensified lignin synthesis and higher expression of phenylalanine ammonia-lyase (PAL) genes (Gayoso et al. 2010).

In lettuce infected with *V. dahliae*, PR-3, PR-5 and putative cysteine protease (LsCP2) genes were identified as expressed only in symptomatic leaves harvested 3 weeks after infection, using suppression subtractive hybridization (Klosterman et al. 2011a).

Differential gene expression analyses on the transcriptional level were also carried out in cotton-*V. dahlia* interactions, and the expression of various genes with implication in resistance was found (Hill et al. 1999; Zuo et al. 2005; Xu et al. 2011a). A recent study by Xu and coworkers (Xu et al. 2011b) using an RNA-seq approach discovered that resistant cotton challenged by *V. dahliae* showed a greater increased expression level of lignin synthesis-related genes and enzyme activity of PAL and peroxidase than susceptible plants as measured at 4, 12, 24 and 48 hpi. Supported by histochemical analysis, they proposed a central role of the lignin metabolism in cotton resistance to *V. dahliae.*

Increased lignification was also found in *Brassica napus* as a defence response to *V. longisporum* (Eynck et al. 2009), and induced peroxidases in Arabidopsis apoplast after infection with *V. longisporum*, as revealed by proteomic and transcriptomic analysis, may have a function in cell wall reinforcement and lignin formation (Floerl et al. 2012). Upregulated endochitinase, a peroxidase, a PR-4 protein and a β-1,3-glucanase were found in apoplast and PR-4 and β-1,3-glucanase in xylem sap of oilseed rape infected with *V. longisporum* (Floerl et al. 2008), while the interaction of *V. longisporum* and *Arabidopsis* showed an increase in six (three peroxidases, serine caroboxypeptidase, α-galactosidase and germin-like protein) apoplastic proteins (Floerl et al. 2012). Massive transcriptional reprogramming involving upregulation of 201 (1 dpi) and 342 (3 dpi) genes belonging to transcriptional factors, stress and defence-related genes and genes involved in secondary metabolism were found as a defence response of Arabidopsis roots to infection with *V. longisporum* (Iven et al. 2012). This in-depth study of genes involved in tryptophan-derived secondary metabolites suggests that these compounds may have an important role in defence against *V. longisporum.*

An intensive defence response was also found in a hop-*V. albo-atrum* compatible interaction through proteomic analysis, in which 252 out of 1,200 2D spots showed infection-specific changes. In the infected roots of the susceptible cultivar, an elevated level of defence-related proteins, such as chitinase, β-glucanase, thaumatin-like protein, peroxidase and germin-like protein, was found, accumulation of which correlated with symptom development. Interestingly though, no infection-specific changes were observed in the roots of the resistant hop cultivar, suggesting constitutive rather than induced resistant mechanisms (Mandelc et al. 2013). We extended these studies to stem tissue in order to monitor changes in hop-*V. albo-atrum* compatible and incompatible interactions on the transcriptional level using cDNA-amplified fragment length polymorphism (AFLP) and annealing control primer (ACP)-based differential display analysis in a time course experiment. The expression pattern of the obtained differential transcript-derived fragments (TDFs) was further examined by reverse-transcription quantitative polymerase chain reaction (RT-qPCR). To support the RT-qPCR expression patterns of TDFs, the plants’ colonization by *V. albo-atrum* was also monitored.

## Material and Methods

### Biological Materials, Plant Inoculation and Sampling

One-year-old potted hop plants multiplied from softwood cuttings of the susceptible variety Celeia and resistant variety Wye Target were artificially inoculated by *V. albo-atrum* isolate-designated T2, which had previously been characterised as lethal pathotype PV1 and genotype PG2 (Radisek et al. 2006). The inoculum was prepared by growing isolate cultures in liquid general fungal medium (Kayser 1992) on a rotary shaker for 5 days at 50 rpm and room temperature in the dark. The conidia were removed from the mycelium by filtration and adjusted to a concentration of 5 × 10^6^ conidia/ml of sterile distilled water by using a Thoma counting chamber (Brand GMBH + CO KG, Wertheim, Germany). Plants of each cultivar were inoculated by dipping the roots in the pathogen inoculum for 10 min. Control plants were similarly mock inoculated with sterile distilled water. After inoculation, the plants were grown as a single bine in a growing chamber (RK-13300, Kambič) under a 12-h photoperiod of fluorescent light (L 58 W/77; Fluora, Osram) at a temperature of 22 °C and relative humidity of 65 % during the light period and 20 °C and 70 % during the dark period.

Three independent inoculation experiments were carried out. (1) Forty-five plants of each cultivar were included in the experiment for bulked samples at 10, 20 and 30 dpi used in differential analysis. (2) For RT-qPCR, four to six plants were sampled at 10, 20 and 30 dpi as biological replications. (3) For measuring fungal colonization, five plants of each cultivar were taken at 3, 6, 10, 15, 20 and 30 dpi. In all three experiments, control non-infected plants were also sampled at each time point. Stem samples were obtained by cutting the basal part of the bines (0–10 cm from the ground) with a sterile scalpel. In the case of the third experiment, samples were taken from the washed roots and two stem sections; bines were cut between ground level and the first node (node 1) and between the first to second node (node 2). All samples were immediately frozen in liquid nitrogen and stored at −80 °C until RNA or DNA extraction. At all sampling time points, the plants were visually assessed for the appearance of foliar symptoms, using a 0–5 scale (Radisek et al. 2003), and the disease severity index (DSI) was calculated according to the Townsend Heuberger formula (Püntener 1981). To confirm the presence of the pathogen in inoculated plants, mycological re-isolation of the pathogen was carried out, except for experiment 3, in which the presence of *V. albo-atrum* was confirmed by qPCR. Only positive samples of inoculated plants were used for further analysis.

### cDNA-AFLP Analysis

Total RNA was isolated from 1 g of frozen hop tissue (−80 °C) using TRIzol® (Invitrogen) reagent according to the manufacturer’s protocol. The precipitated (isopropanol, 3 M sodium acetate pH 5.2) and washed (70 % ethanol) RNA was re-suspended in RNAse free water and frozen at −80 °C until further use. The concentration and purity of RNA was determined by means of spectrophotometry and its integrity by agarose gel electrophoresis.

Messenger RNA (mRNA) was isolated from 100 to 1,000 μg of total RNA using the PolyATtract® mRNA Isolation System, which is based on streptavidin paramagnetic particles-biotin affinity, according to the supplier’s protocol. The captured mRNA was concentrated by precipitation (isopropanol, 3 M sodium acetate pH 5.2), dissolved in a small amount of RNAse free water and its concentration measured by spectrophotometer. The mRNA (2 μg) was reverse transcribed to cDNA by the Universal RiboClone® cDNA Synthesis System (Promega) according to the supplied protocol. Precipitated (2.5 M sodium acetate, ethanol) and washed (70 % ethanol) cDNA was re-suspended in 25 μl of TE (10 mM Tris-HCl, 1 mM EDTA, pH 8.0) and the concentration measured by spectrophotometer. Samples were stored at −20 °C until further use.

The original AFLP protocol (Vos et al. 1995) was used on prepared cDNA with the exchange of six-cutter *EcoR*I for *Pst*I enzyme. Five hundred nanograms of cDNA was restricted; adapters were ligated to the ends of the fragments and pre-amplified with primers harbouring A and C selective nucleotides. Selective amplification reactions were prepared by combining 3 *Pst* (P) and 8 *Mse* (M) primers with 2 or 3 selective nucleotides, yielding 24 different combinations (P-ACA, P-AGA, P-AAC and M-CG, M-CT, M-CTG, M-CAG, M-CTA, M-CA, M-CC, M-CTC). *Pst* primers were 5′ end labelled with CY5 dye, enabling us to use fluorescent detection and analysis on an ALFExpressII automated system according to the published protocol (Gril et al. 2008).

Combinations showing differentially expressed fragments were amplified with unlabelled *Pst* primers, using the same conditions and analysed on a vertical S2 manual denaturing electrophoresis system (Whatman) and detected using the silver staining protocol (Jakse et al. 2001). Differentially expressed cDNA fragments were re-amplified using AFLP primers from silver staining gels (Jakse et al. 2004). Re-amplified PCR products were resolved on 1.2 % agarose gel, excised and cleaned by the silica glass milk protocol using a Silica Bead DNA Gel Extraction Kit (Fermentas).

### ACP Differential Display

Total RNA was isolated from 100 mg of frozen plant tissue (−80 °C) using a Spectrum Plant Total RNA Kit (Sigma-Aldrich) according to the manufacturer’s protocol. The concentration and purity of total RNA was determined by means of spectrophotometry and its integrity by agarose gel electrophoresis. A commercialized ACP system (Kim et al. 2004) technology implemented in a GeneSnare Differential Expression Kit (Sigma-Aldrich) was used for differential display. All steps were carried out according to the supplied protocol. In the first step, mRNA from the total RNA sample (4 μg) was reverse-transcribed into single-stranded cDNA, which was used in subsequent PCR cycles as a template for differential display. In the PCR reaction, 1 μl of cDNA was amplified by ACP-arbitrary primer and polyT-ACP primer. Altogether, 24 different ACP-arbitrary primers were used. Amplification reactions were resolved on 1.2 % agarose gels. Differentially expressed GeneSnare DNA fragments (presence-absence) were excised from the gel and cleaned by the silica milk protocol using a Silica Bead DNA Gel Extraction Kit (Fermentas).

### Cloning and Sequencing of Differentially Expressed cDNA-AFLP and GeneSnare DNA Fragments

Cleaned DNA fragments (cDNA-AFLP or GeneSnare) were cloned into a pGEM T-easy vector system (Promega). At least five white bacterial colonies from each cloning reaction were tooth-picked into 50 ul of TdE (10 mM Tris-HCl, 0.1 mM EDTA, pH 8.0), boiled for 8 min, and 5 μl was PCR amplified with vector specific T7 and SP6 primers. PCR products were cleaned with an ExoSAP-IT mixture of exonuclease and shrimp alkaline phosphatase (USB Biochemicals). Cleaned fragments were sequenced from both directions using BigDye Terminator v3.1 sequencing chemistry and analysed on a 3730XL Applied Biosystems DNA Analyzer.

### Sequence Editing and Bioinformatics

Sequencing chromatograms were edited and assembled using Codon Code Aligner v. 2.0.6 (CodonCode Corporation). All vector and primer specific sequences were removed, and sequences were assembled in contigs of at least 90 % identity and a minimum of 35 bp of overlap. All sequences shorter than 70 bp were removed from the project.

Assembled contigs and singleton sequences were searched using the BLASTN and BLASTX algorithm (Altschul et al. 1990) against the NCBI nucleotide collection (nr/nt), non-human, no-mouse ESTs (est_others) and non-redundant protein databases. In addition, two local nucleotide blast databases were formatted using a stand-alone blast package harbouring DNA sequences of PlantGDB Assemblies for *H. lupulus* (Dong et al. 2004) and a genome sequence of *V. albo-atrum* VaMS.102 (Klosterman et al. 2011b). The first two databases were searched using the BLASTX algorithm, allowing comparison of nucleotide sequences against the protein database, and the BLASTN algorithm against *Humulus lupulus* PlantGDB and the *V. albo-atrum* genome sequence. The significance of the results was filtered at an e value lower than 10^−5^.

The Blast2GO software package (Conesa et al. 2005) was used for gene annotation to reveal the gene ontology terms for the query sequences of cDNA-AFLP and GeneSnare transcripts. Terms describe their biological processes, molecular function and cellular components.

### Real-time PCR Analysis

Real-time PCR was performed using Fast SYBR Green technology on an ABI PRISM 7500 Fast Sequence Detection System (Applied Biosystems, Foster City, USA). A master mix for each PCR run was prepared with SYBR Green PCR Core Reagents (Applied Biosystems, Foster City, USA). The final reaction, in a total volume of 20 μl, was as follows: 10 μl fast SYBR Green master mix, 10 ng of cDNA or 50 ng of DNA in the case of fungi quantification and 300 nM of each specific forward and reverse primer. The following amplification program was used: 95 °C 20 s, 40 cycles at 95 °C for 3 s followed by 60 °C for 30 s. All samples were amplified in three technical replicates acquired from the same cDNA/DNA sample. The amplification levels of genes were determined as Ct (cycle threshold) values, which represent the number of cycles needed to reach a threshold of amplification fixed in the exponential phase of the PCR reaction (Walker 2002). The amplification efficiency was calculated for each amplified target sequence with ABI 7500 software (version 2.0.4), based on the slope of the standard curve. Amplified products were verified on a 4 % agarose gel. All corresponded to the expected sizes. Melting curve experiments determined the melting temperatures of the target nucleic acid sequences and identified no non-specific PCR amplifications, showing a single amplified product for all genes.

### qPCR of Fungal DNA

DNA from infected and control plants was extracted as described by Kump and Javornik (1996). The amount of *V. albo-atrum* DNA in infected plants was determined with real-time PCR from a calibration curve using six 5-fold serial dilutions of *V. albo-atrum* genomic DNA, ranging from 150 to 0.05 ng/μl. PCR assays were validated for the influence/inhibition of fungi DNA amplification in the presence of plant DNA. The experiment was performed by mixing 5 and 500 pg/μl of pathogen DNA (*V. albo-atrum*) with 5 and 50 ng/μl of healthy hop plant DNA. The results showed that the amplification of fungal DNA is not influenced by the plant matrix. Normalization was performed by using two plant reference genes, CAC and SAND, as previously described in Stajner et al. (2013). The absence of fungi in the control plants was also confirmed by real-time PCR.

### Two Step RT-qPCR of TDFs

Single-stranded cDNA was synthesized from 1 μg of total RNA using a High Capacity cDNA Reverse Transcription Kit (Applied Biosystems, Foster City, USA). Serial standard dilutions in a range of 50, 12.5, 3.12, 0.8 and 0.2 and 0.05 ng obtained from pooled cDNA samples were used to check the real-time PCR efficiency and to obtain a standard curve. Expression was determined using the ΔΔCt method. Transcript abundances derived from four to six biological replicates were normalized to SAND and CAC reference genes (Stajner et al. 2013), and fold differences were standardized to control (non-infected samples) expression values. Fold differences were transformed by using a binary logarithm (log2).

### Primer Design

Primer sequences for selected TDFs obtained by cDNA-AFLP and GeneSnare analysis were designed by using Primer Express 3.0.0 Applied Biosystems software (Supplemental Table S1). Fungal DNA was detected and quantified with primer pair 9-1gs-F-GGTAACGTCATCGAACGACATC and 9-1gs-R-CACACGCTACATATCAAACAGCATAT (Radisek et al. 2004). Primer pairs for reference genes SAND and CAC, which served as endogenous controls, were used as previously described in Stajner et al. (2013).

## Results and Discussion

### Plant Inoculation and Colonization

After inoculation, plants were assessed for the first foliar wilt symptoms, which appeared on the susceptible variety Celeia at 20 dpi at the level of 30 % of DSI. The symptoms progressed and became more severe at 30 dpi (53.8 % of DSI). No visual symptoms were observed on the resistant Wye Target plants or mock-inoculated control plants (Supplemental Text S1). The successful infection of inoculated plants was confirmed by pathogen re-isolation analysis, which at 30 dpi revealed 100 and 82 % infected plants of susceptible Celeia and resistant cultivar Wye Target, respectively.

**Fig. 1:**
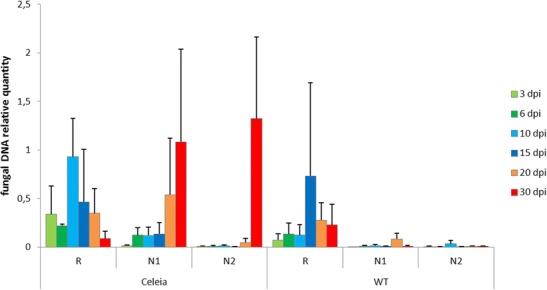
Quantification of fungal biomass in roots (*R*) and two stem sections (*N*1, *N*2) of susceptible (*Celeia*) and resistant (*Wye Target*) cultivar over the time course post inoculation showed increased biomass of fungus in stem sections of susceptible plants. In resistant plants, the fungus was restricted to the roots, with a peak at 15 dpi

The colonization dynamics of *V. albo-atrum* in susceptible and resistant hops was further examined by measuring the amount of fungal DNA by qPCR in roots and two stem sections at six time points (Fig. 1). In the susceptible cultivar, a cyclical pattern of colonization was observed in roots, with two peaks of fungal population, at 3 and 10 dpi. The highest amount of fungal DNA was detected at 10 dpi, followed by steady decrease of fungus until 30 dpi. In the stem, the fungal biomass increased through time without intermittent decrease. The lower section below the first node was colonized first, followed by the upper section, indicating a continuous spread of the fungus up the stem. In the resistant cultivar, root colonization was less extensive than in the susceptible cultivar and was delayed, with a fungal population peak at 15 dpi. Fungal DNA was detected in stem sections of the resistant cultivar but remained at very low levels throughout the experiment. No fungal DNA was detected in control samples from non-inoculated plants. The colonization pattern thus showed a continuous growth of fungus in the roots of susceptible and resistant cultivars up to 10 and 15 dpi, respectively, followed by fungal decline in both cultivars, probably due to plant resistant responses. The fungus then overcame the resistance and recovered in the susceptible cultivar, with continuous colonization of the stem, whereas its further growth in the stem of the resistant cultivar was very restricted. Non-specific plant responses to fungus colonization in the form of physical obstructions were also observed in the hop-Verticillium system, as extensive, very early tylosis formation and the synthesis of coating materials around infected vessels in susceptible plants, while resistant plants showed less tylosis throughout the plant and no coating response (Supplemental Text S2). A restriction of fungus or lower amount of fungus in resistant versus susceptible cultivars has also been shown in other host-Verticillium studies, such as in oilseed rape, olive, lettuce and cotton, by measurement of fungal DNA during colonization, as well as physical responses in terms of cell wall thickening, lignin deposition and phenolic compound synthesis (Eynck et al. 2007; Markakis et al. 2009; Zhang et al. 2013; Vallad and Subbarao 2008), reflecting the specifics of the host-Verticillium interaction.

### Isolation of Differentially Expressed TDFs and Sequence Analysis

Changes in the hop transcriptome from early colonization to symptom development after inoculation with *V. albo-atrum* were analysed by differential analysis using cDNA-AFLP and ACP RT-PCR. A comparison of transcript expression was made between infected and mock-inoculated plants of susceptible cultivar Celeia (compatible interaction) and resistant cultivar Wye Target (incompatible interaction). Samples of each treatment at 10, 20 and 30 dpi were bulked and used for RNA extraction. For RT-qPCR analysis, samples were taken at each time point in four to six biological replications.

In cDNA-AFLP analysis, TDFs were selected on the basis of differentially displayed TDFs between control and infected samples at the same or different time points for compatible and incompatible interactions. Around 2,500 bands, ranging in size from 50 to 750 bp, were amplified by selected primer combinations, of which 257 TDFs showed altered expression patterns. Of the differentially expressed TDFs, 117 (45.5 %) were successfully re-amplified, cloned and sequenced, obtaining in total 90 contigs and 43 singletons for sequence analysis. These 134 sequences, with an average size of 137 bp, were subjected to sequence analysis. Fifty six and 21 TDFs were isolated from susceptible and resistance cultivars, respectively, as induced after inoculation, and 18 TDFs were induced in both analysed cultivars. The remaining sequences showed altered expression either in control or inoculated plants. The total number of TDFs and the number of cloned TDFs is similar to the published cDNA-AFLP analysis of compatible and incompatible interactions. Using cDNA-AFLP, Sestili et al. (2011) studied interactions between melon and *Fusarium oxysporum* f.sp. *melonis*, and they successfully sequenced 53 % of differentially expressed transcripts, while only 16 % (Dhariwal et al. 2011) and 11 % (Wang et al. 2010) of TDFs were successfully sequenced in studies of interactions between wheat-leaf rust and wheat-stripe rust fungi, respectively.

In ACP-based differential display RT-PCR analysis, 24 different ACP-arbitrary primers were first tested, of which 12 generated TDFs in the range from 200 to 800 bp. Sixty-six differentially expressed TDFs were re-amplified, cloned and sequenced, giving a total of 84 sequences, comprising 31 contigs and 53 singletons, with an average size of 440 bp. Twenty-two TDFs were upregulated in infected susceptible Celeia, 14 in resistant Wye Target and 24 in both infected cultivars, and the remaining TDFs were isolated from control samples. ACP systems have been widely used in differential gene expression studies since the development of improved systems and commercially available kits (Kim et al. 2004). Although more studies have been reported for animals, the ACP differential display has been successfully used for identification of genes involved in spine formation on seeds of carrots (Park et al. 2006), Cd-responsive genes in *Solanum nigrum* (Xu et al. 2009), drought induced genes in barley (Lee et al. 2011) and induced genes in cotton after inoculation with *Aspergillus flavus* (Lee et al. 2012). We found ACP-based differential display more advantageous than cDNA-AFLP because of its methodological simplicity, due to the use of agarose gels instead of long AFLP polyacrylamide gels and due to obtaining much longer TDFs with good matches to known sequences.

In both differential analyses, a much higher number of TDFs was isolated from the inoculated susceptible cultivar than from the inoculated resistant cultivar, indicating stronger transcriptomic changes in the compatible interaction. Similar responses have been observed in hop roots challenged with *V. albo-atrum* as high infection-specific changes in protein abundance in a compatible but not in an incompatible interaction (Mandelc et al. 2013). In the tomato-Verticillium pathosystem, more intensive transcriptional changes were also found in the compatible interaction (Robb et al. 2007; van Esse et al. 2009), as well as on the root proteome level (Robb et al. 2012), in which only a modest response by the resistant cultivar was observed in comparison to the susceptible one. Similar intensities in responses were also observed in interactions between melon and the vascular pathogen *F. oxysporum* f.sp. *melonis* (Sestili et al. 2011). Intensive transcriptional and proteomic changes in compatible interactions and lack of such changes in incompatible interactions have been interpreted as due to disease wilting symptoms, perhaps as a consequence of an active (exaggerated) plant response (Sestili et al. 2011; Robb et al. 2012) or resistance might be due to the constitutive presence of antifungal substances (Mandelc et al. 2013).

Two hundred and seventeen TDFs derived from the two analyses were subjected to sequence analysis using BLASTX and BLASTN comparisons to databases augmented with further Blast2GO annotation. BLASTN (e < 10^−5^) and BLASTX (e < 10^−10^) analyses revealed hits to known sequences for 184 TDFs (120 for cDNA-AFLP and 64 for ACP sequences), while 33 TDFs showed no hits to database nucleotide or protein sequences at defined thresholds. In BLASTN comparison, 138 TDF sequences revealed hits over >80 % of the query length, while BLASTX searches combined with Blast2GO annotation analysis resulted in 92 positive matches. Out of 92 known proteins, 84 belonged to plant proteins and eight TDFs to fungal protein sequences (Supplemental Table S2). The hop TDFs homologous to plant genes were categorized by gene ontology according to biological process, molecular function and cellular components (Table 1). Further Blast2GO annotation was able to assign gene ontology terms to 64 TDFs (29.5 %). Biological process ontology was successfully assigned to 58 sequences, molecular function ontology to 55 and cellular components ontology to 51 sequences (Table 1 and Supplemental Fig. S1).

**Table 1:** Assigned gene ontology classes for biological processes, molecular function and cellular components to TDFs

Biological process (58)	Molecular function (55)	Cellular components (51)
Signalling (7) Rhythmic process (1) Response to stimuli (25) Reproduction (6) Metabolic process (50) Biological regulation (19) Cellular component organisation or biogenesis (19) Cellular process (53) Developmental process (12) Growth (5) Immune system process (3) Localization (21)	Transport activity (8) Structural molecule activity (5) Molecular transduction activity (1) Electron carrier activity (4) Catalytic activity (37) Binding (36)	Symplast (5) Organelle (42) Membrane-enclosed lumen (5) Membrane (31) Cell (49) Cell junction (5) Extracellular matrix (1) Extracellular region (7) Macromolecular complex (17)

Twenty-nine plant TDFs were selected on the basis of their gene expression patterns and similarity to known genes implicated in the response to stimuli, transport activity, binding and membrane localization, in order to examine their expression profiles by RT-qPCR during the infection process. Selected TDFs are designated in Supplemental Table S2. Twelve TDFs that showed upregulation only in the resistant cultivar or in both resistant and susceptible cultivars are interpreted in the following sections. One of the TDFs matched the PR protein acidic endochitinase, and in order to monitor the expression of other PR genes in hop-Verticillium interactions, four PR genes (PR1, PR-2, PR-3 and PR-5) were additionally tested. The remaining 15 TDFs analysed by RT-qPCR showed either non-distinctive expression patterns between infected and mock-inoculated plants or between resistant and susceptible plants, or we could find no reasonable explanation for their role in compatible or incompatible interactions (Supplemental Fig. S2).

Eight isolated TDFs (Supplemental Table S2) showed similarities to fungal proteins. Two TDFs were highly similar to the lectin and hypothetical protein, respectively, in the annotated *V. albo-atrum* VaMs102 genome (Klosterman et al. 2011a), and the other six TDFs matched proteins in *Fusarium graminearum* (2TDFs), *Auricularia delicate* (2TDFs), *Setosphaeria turcica* (1 TDF) and *Zymoseptoria tritici* (1TDF), with similar proteins encoded either in *V. albo-atrum* VaMs102 or *V. dahilae* VdLs.17 genomes (Klosterman et al. 2011b). The number of Verticillium-derived TDFs is lower than expected, since the fungus was present in the inoculated hop plants, as shown by the colonization pattern (Fig. 1) and the re-isolation tests. It seems that the ratio between fungus and plant mRNA favours plant mRNA and that the methods used were unable to detect more fungal TDFs. However, in several other differential expression analyses of the Verticillium-host system, no fungus sequences were reported (Robb et al. 2009; Robb et al. 2012; van Esse et al. 2009). *In planta* detection of fungal protein might suggest their role in fungal virulence and thus make the eight TDFs potential candidate genes to be tested for their function. The dynamics of lectin expression of the *V. albo-atrum* TDFs was further examined by RT-qPCR.

### Fungal TDF, Quantification of *V. albo-atrum* Lectin in Susceptible and Resistant Hop Cultivars

**Fig. 2:**
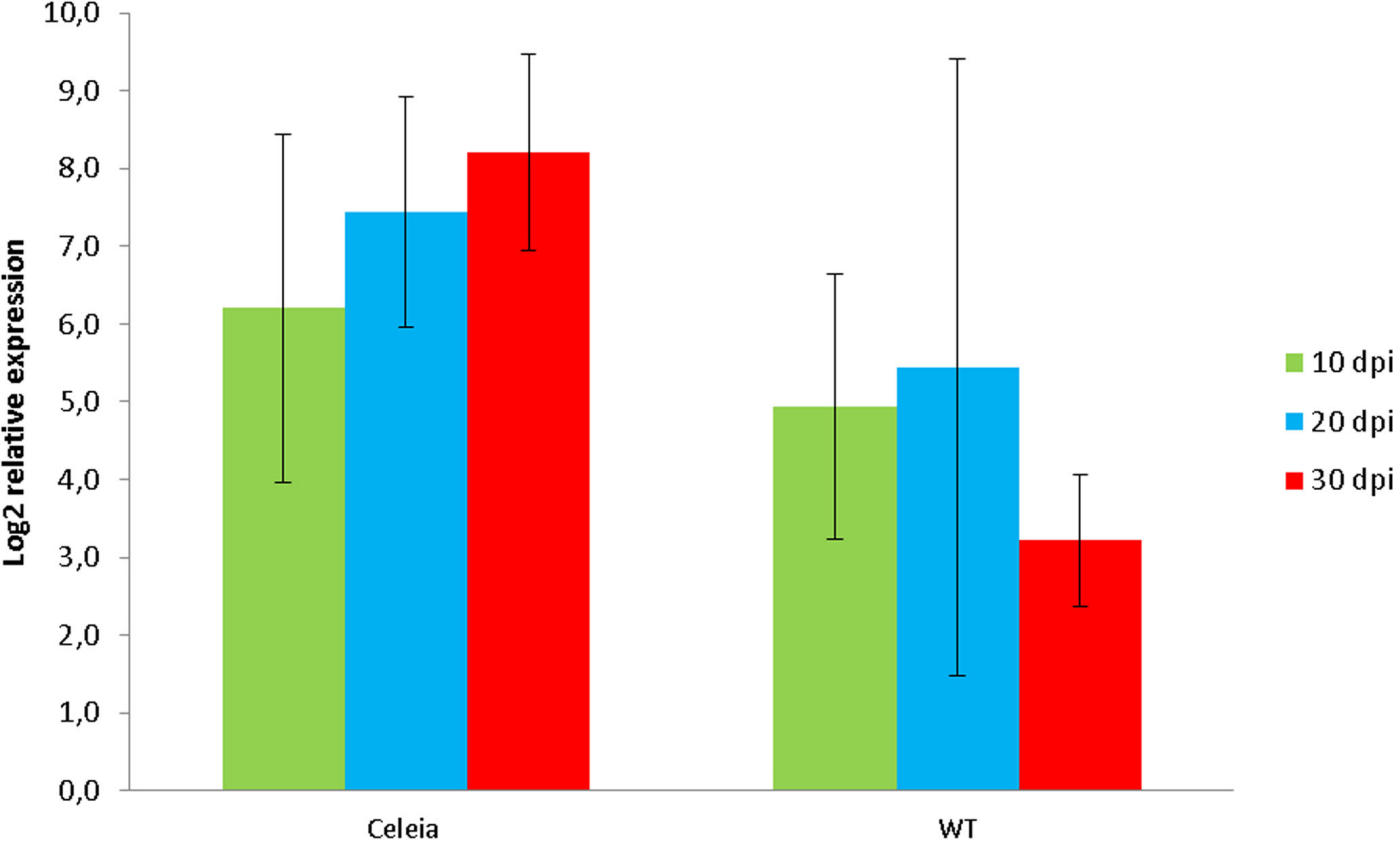
Relative expression level of *V. albo-atrum* lectin gene *in planta* is increasing in susceptible cultivar Celeia and decreasing in resistant cultivar Wye Target (*WT*) over the time course. *Error bars* indicate SD of five biological replicates

TDF HO059232 matched the *V. albo-atrum* VaMs.102 lectin gene with 98 % identity. A Blast search of the *V. dahliae* genome and some other related fungal genomes (results not shown) showed low-identity matches, indicating that the *in planta* identified lectin might be specific to the species *V. albo-atrum.* The RT-qPCR expression pattern of the lectin gene showed lower expression in the resistant than in the susceptible cultivar (Fig. 2), and a steady increase from 10 to 30 dpi in the compatible interaction, while an increase from 10 to 20 dpi followed by decline to the last time point was observed in the incompatible interaction. RT-qPCR expression patterns of HO059232, resembling a colonization pattern (Fig. 1), suggest that the fungus had spread through the susceptible plant unimpeded but had been arrested in the resistant cultivar around 20 dpi, probably due to the activation of a strong defence response. The *in planta* identified lectin with chitin-binding activity belongs to carbohydrate-binding module family 18, CBM18 (Wright et al. 1991). This chitin-binding domain is found in one or more copies in plant and fungal proteins that are involved in recognition or binding of chitin oligomers (Lerner and Raikhel 1992). Whether this lectin is implicated in any way in *V. albo-atrum*, virulence would be interesting to examine, since one of the known effector proteins, Ave4 from *Cladosporium flavum*, is a related chitin-binding lectin (CBM14) that functions as a defensive virulence factor, protecting the fungal cell wall against plant chitinases by binding to chitin (van den Burg et al. 2006; Stergiopoulos et al. 2010).

### Hop TDFs induced in compatible and incompatible interactions are involved in stress-related proteins (PR proteins, aconitase, epoxy hydrolase), morphogenesis (Cobra protein), transport (multidrug and toxic compound extrusion (MATE) protein) and protein-protein interactions (14-3-3 protein)

**Fig. 3:**
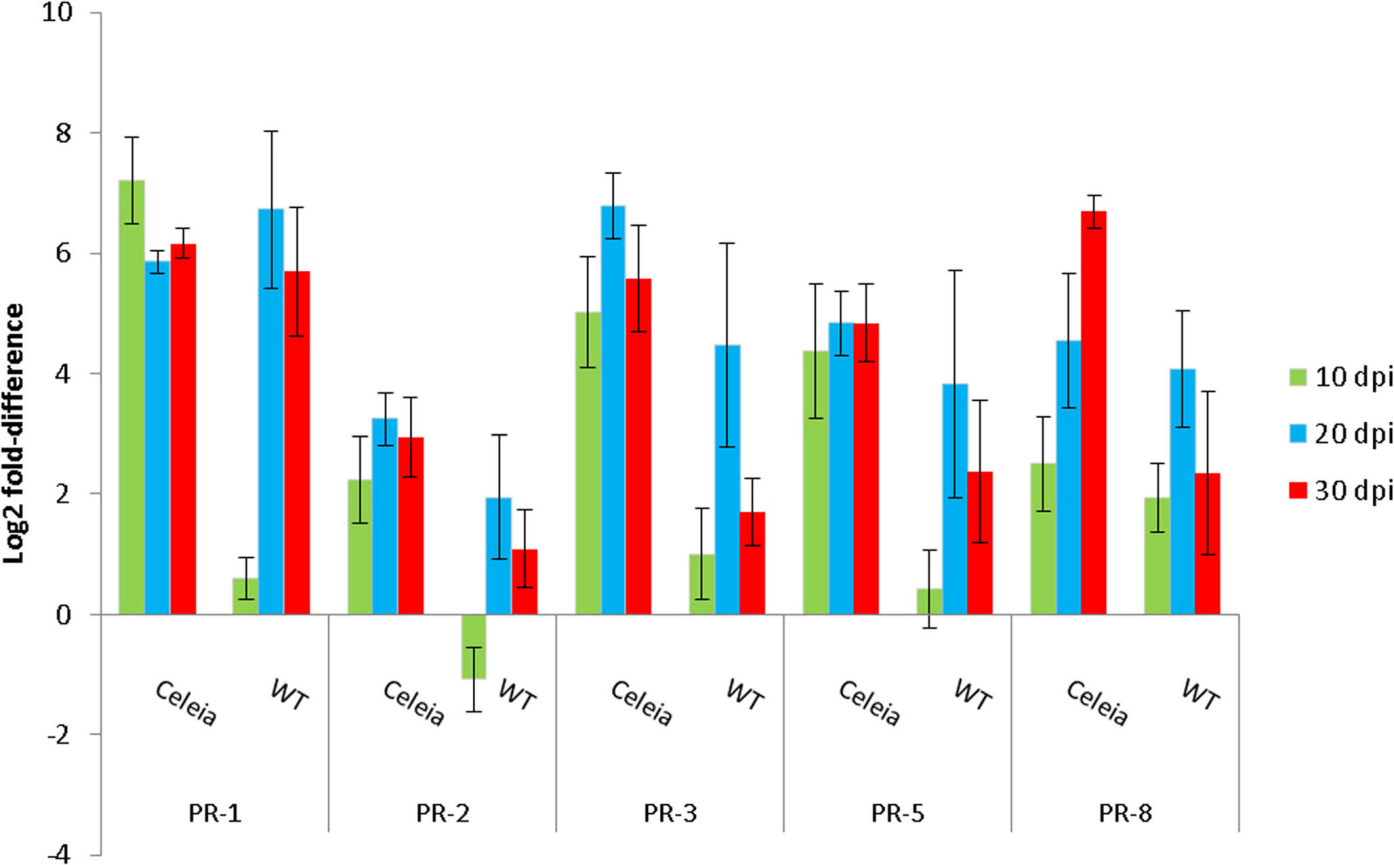
RT-qPCR relative expression levels of PR-1, β-1,3-glucanase (*PR*-2), chitinase (*PR*-3), thaumatin-like protein (*PR*-5) and endochitinase (PR-8; HO059226) in susceptible Celeia and resistant Wye Target (*WT*) cultivars infected with *V. albo-atrum*. The values are relative to the level of expression in mock-inoculated plants at 10, 20 and 30 dpi. *Error bars* indicate SD of five biological replicates

One TDF (HO059226) showed very high similarity to hevamine (Rozeboom et al. 1990), a plant defence protein with chitinase and lysozyme activity (Vanscheltinga et al. 1994). Hevamine belongs to a class III endochitinase, which is classified into the pathogenesis-related protein family PR-8 (Ferreira et al. 2007). It hydrolyses chitin and peptidoglycan and thus provides an important defence against pathogenic fungi. The RT-qPCR pattern (Fig. 3) showed induction of hevamine in both interactions, with differences over the time course. In the susceptible cultivar, the increase was stronger and gradual, while in resistant plants, the peak expression was found at 20 dpi followed by downregulation. Since differential expression analysis revealed only hevamine of typical PR proteins (van Loon et al. 2006), we also examined PR-1, chitinase (PR-3), β-1,3-glucanase (PR-2) and a thaumatin-like protein (PR-5), which showed significant upregulation in hop root proteome in the compatible interaction (Mandelc et al. 2013). All four analysed genes were highly upregulated in the compatible interaction (Fig. 3). The most dramatic increase compared to mock-inoculated plants was observed for PR-1 at 10 dpi, followed by a decrease at later time points. PR-1, chitinase, β-1,3-glucanase and thaumatin-like protein reached a peak level at 20 dpi in the incompatible interaction, but the response in resistant plants was weaker than in the compatible interaction. For example, in susceptible plants, upregulation of PR-1 was 153-fold, while in resistant plants only 1.5-fold at 10 dpi.

Synthesis of PR proteins is induced in various host-Verticillium interactions. Infection of cotton with *V. dahlia* or its elicitor induced expression of chitinase and β-glucanase, both fungal cell wall degrading enzymes (McFadden et al. 2001; Dubery and Slater 1997). Endochitinase (PR-3) and β-1,3-glucanase (PR-2) were also induced, together with peroxidase and PR-4 protein, in leaf apoplast of oilseed rape infected with *V. longisporum* (Floerl et al. 2008). PR-5 and PR-10 were identified as differentially expressed PR genes in another cotton-*V. dahliae* study (Xu et al. 2011b), and PR-3 and PR-5 were found only in lettuce leaves infected with *V. dahliae*. A differential gene expression study of tomato-Verticillium systems identified induced PR-1, PR-4 and PR-5 (van Esse et al. 2009) and, in another study, PR-1, PR-2, PR-3, PR-9 and PR-10 (Robb et al. 2012). In hop-Verticillium interactions, a much higher induced level of PR1, PR-2, PR-3, PR-5 and PR-8 genes was observed in the compatible interaction, which correlated with the development of disease symptoms and thus suggests that some of the disease symptoms might be the consequences of the action of PR proteins, as noted by Robb et al. (2012) and Mandelc et al. (2013). In resistant hop plants, in which the fungus is limited to the roots and the base of the stem (Fig. 1), systemic signalling induces synthesis of PR proteins with a similar expression pattern, i.e., a slight increase at 10 dpi, reaching peak expression at 20 dpi and then a decline towards 30 dpi, suggesting that the fungus is progressing and plant defence is building up towards 20 dpi, and at 20 dpi, the arms race changes course, with a strong plant defence response followed by fungus elimination (Fig. 1).

**Fig. 4:**
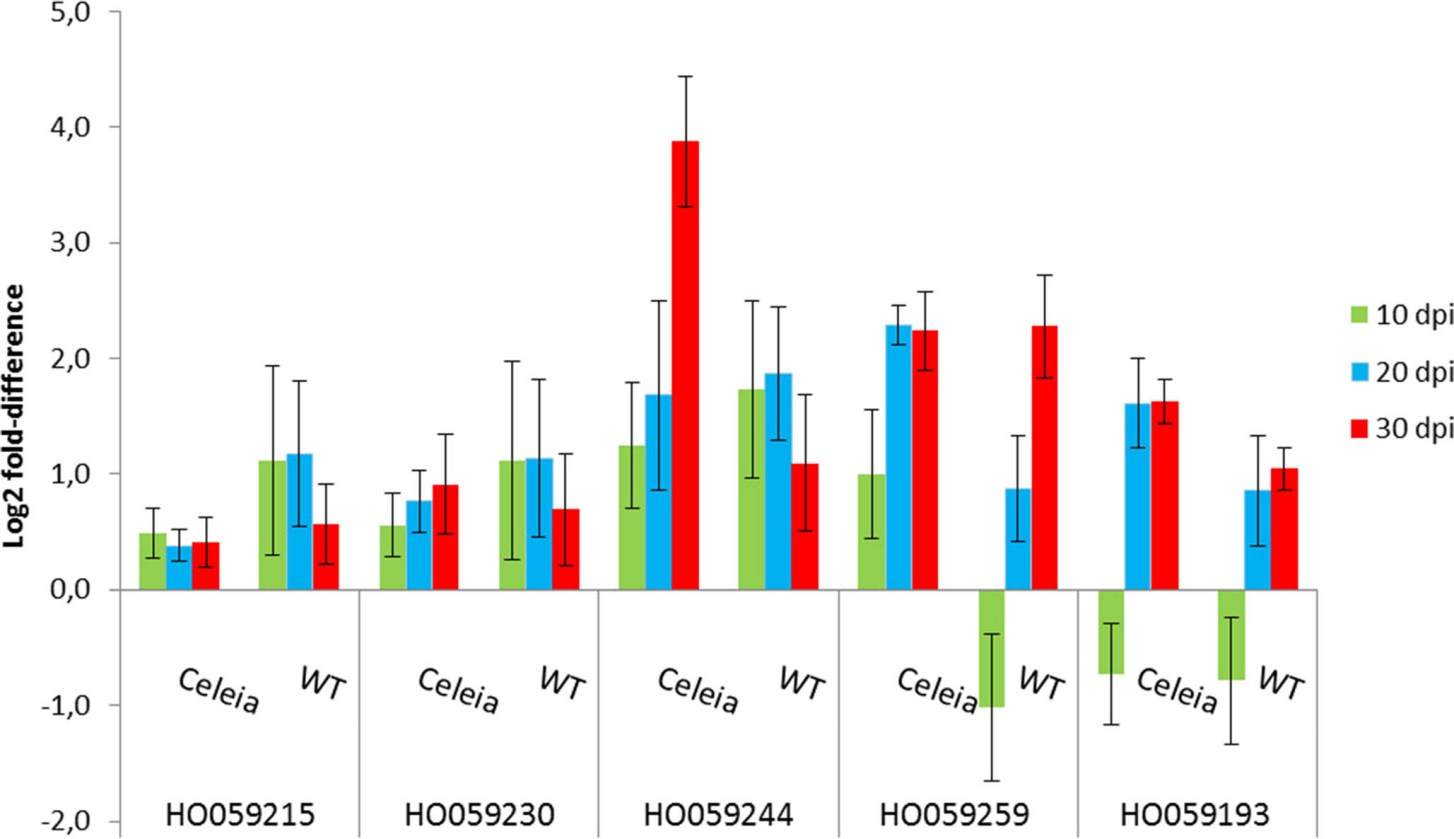
RT-qPCR relative expression levels of TDFs HO059215 (Aconitase), HO059230 (Epoxy hydrolase), HO059244 (MATE-like protein), HO059259 (Cobra-like protein) and HO059193 (14-3-3 protein) in susceptible Celeia and resistant Wye Target (*WT*) cultivars infected with *V. albo-atrum*. The values are relative to the level of expression in mock-inoculated plants at 10, 20 and 30 dpi. *Error bars* indicate SD of five biological replicates

HO059215 transcript matched cytoplasmic aconitase (CAN62964). Measurements of the expression after infection showed a response to infection at 10 and 20 dpi, then a slight decrease in the resistant cultivar, in contrast to the compatible interaction, in which a similar elevated expression was detected at all three time points (Fig. 4). The question arises of whether an increased level of aconitase in the incompatible interaction has any role in resistance, since very little is known about the role of plant aconitases in defence mechanisms. Cytosolic and mitochondrial aconitases are recognized in plants, which catalyse isomerization of citrate to isocitrate in the tricarboxylic acidcycle. The RNA-binding property of Arabidopsis aconitase has been shown by its binding to 5′UTR of the superoxide dismutase (CSD2) transcript (Moeder et al. 2007). In addition, based on experiments with silenced aconitase in Arabidopsis and *Nicotiana benthamiana* plants, the same authors suggest an active role of aconitase in activation of a hypersensitive response (HR) and cell death associated with pathogen infection. The induced level of aconitase in the hop-Verticillium incompatible interaction cannot be related to HR, because of its absence in vascular diseases, so the elevated level of aconitase in the resistant cultivar after infection with fungal pathogen remains to be explored.

TDF HO059230, with a similarity to predicted epoxy hydrolase 2 (EH 2) in wild strawberry (*Fragaria vesca* subsp. *vesca*), also showed induced expression up to 20 dpi in the incompatible interaction and a gradual increase in the compatible interaction (Fig. 4), implicating this enzyme in the response to vascular infection. This enzyme degrades reactive epoxides, oxylipins generated from unsaturated fatty acids by lipoxygenases and peroxygenase, to dihydrodioles, which can be transformed into compounds acting in signalling, wound or antimicrobial responses and as monomers in cutin synthesis (Blee 2002). An increased level of EH has been found in a few cases in response to pathogen attack, such as in rough lemon (*Citrus jambhiri*, RlemEH) after infection with *Alternaria alternate* (Gomi et al. 2003) or in some interactions of tobacco with tobacco mosaic virus (TMV), *Pseudomonas syringae* pv. *syringae*, *P. syringae* pv. *tabaci* and *Agrobacterium tumefaciens*, as well as with non-plant pathogens *Escherichia coli* and *Pseudomonas fluorescens* (Szatmari et al. 2006). In tobacco, the authors interpreted the induction of EH 1 as a coincidental event with the development of basal defence.

Induced expression in the compatible interaction was also found for TDF HO059259 (Fig. 4) with a sequence similarity to a Cobra-like protein in soybean and to Arabidopsis Cobra-like protein at locus at3g02210. Cobra-like proteins are essential for cellulose biogenesis, through its function in cellulose microfibril orientation (Roudier et al. 2005; Liu et al. 2013), thus being important for biosynthesis of cell wall constituents. The induced expression of the Cobra gene in our compatible as well as in incompatible interaction might suggest intensive cellulose deposition or cell wall formation as a response to fungal infection.

Increased expression in both interactions, although higher in susceptible hop at 30 dpi, also showed sequences homologous to a MATE-type protein (Fig. 4). MATE-type proteins belong to a large multigene family in plants. The well-described MATE transporter FRD3 controls iron deficiency responses (Rogers and Guerinot 2002), and EDS5 is a component of salicylic acid-dependent signalling for disease resistance in Arabidopsis and functions as MATE transporter in the export of SA from the chloroplast to the cytoplasm (Nawrath et al. 2002). Our TDF HO059244 (Fig. 4), with similarity to XP_003634196, *Vitis vinifera* MATE efflux family protein 6-like was induced in both cultivars at 10 dpi, after which the expression rose steadily in the susceptible and slowly decreased in the resistant cultivar. The best match of XP_003634196 to Arabidopsis MATE proteins were for At1g73700 and At2g34360 entries, to which a specialized function has not yet been assigned. However, strong induction of a MATE-like protein in hop-Verticillium interactions suggests that this protein may have a role in the plant-fungal interaction or in vascular diseases.

TDF HO059193, with a high similarity to cotton 14-3-3 protein (ABY65004), was downregulated at 10 dpi in both interactions, followed by increased expression at 20 and 30 dpi in both cultivars, although stronger in the compatible interaction (Fig. 4). An elevated level of 14-3-3 protein was also found in a cotton-Verticillium interaction (Hill et al. 1999) and downregulated in a tolerant tomato-Verticillium interaction (Robb et al. 2007). 14-3-3 proteins are a family of conserved regulatory proteins that function by binding to diverse proteins to modulate their function in many biological processes. They have been implicated in disease resistance as proteins that promote resistance by direct binding with R proteins, as shown for Arabidopsis RPW2.8 resistant gene conferring resistance to fungal pathogens of *Golovinomyces* spp. (Yang et al. 2009) and N protein from tobacco resistant to tobacco mosaic virus (Ueda et al. 2006), or by enhancing signalling cascade and subsequently positively regulating immunity-associated programmed cell death, as shown in a tomato interaction with *P. syringae* pv. *tomato* (Oh and Martin 2011; Oh et al. 2010). The above cases demonstrate a specific role of 14-3-3 proteins in plant immunity, although each organism has multiple 14-3-3 isoforms, which can interact with many target proteins. Our detection of increased expression of hop 14-3-3 after infection is therefore another indication of the involvement of these proteins in plant immunity.

### Hop TDFs upregulated in the incompatible interaction and downregulated in the compatible interaction include proteins involved in ubiquitination (SKP1), vesicle trafficking (cdc48), protein degradation (puromycine-sensitive amniopeptidase), protein-protein interactions (syntaxin and Fk506), transport (acyl-CoA-binding protein) and morphogenesis (furry protein)

**Fig. 5:**
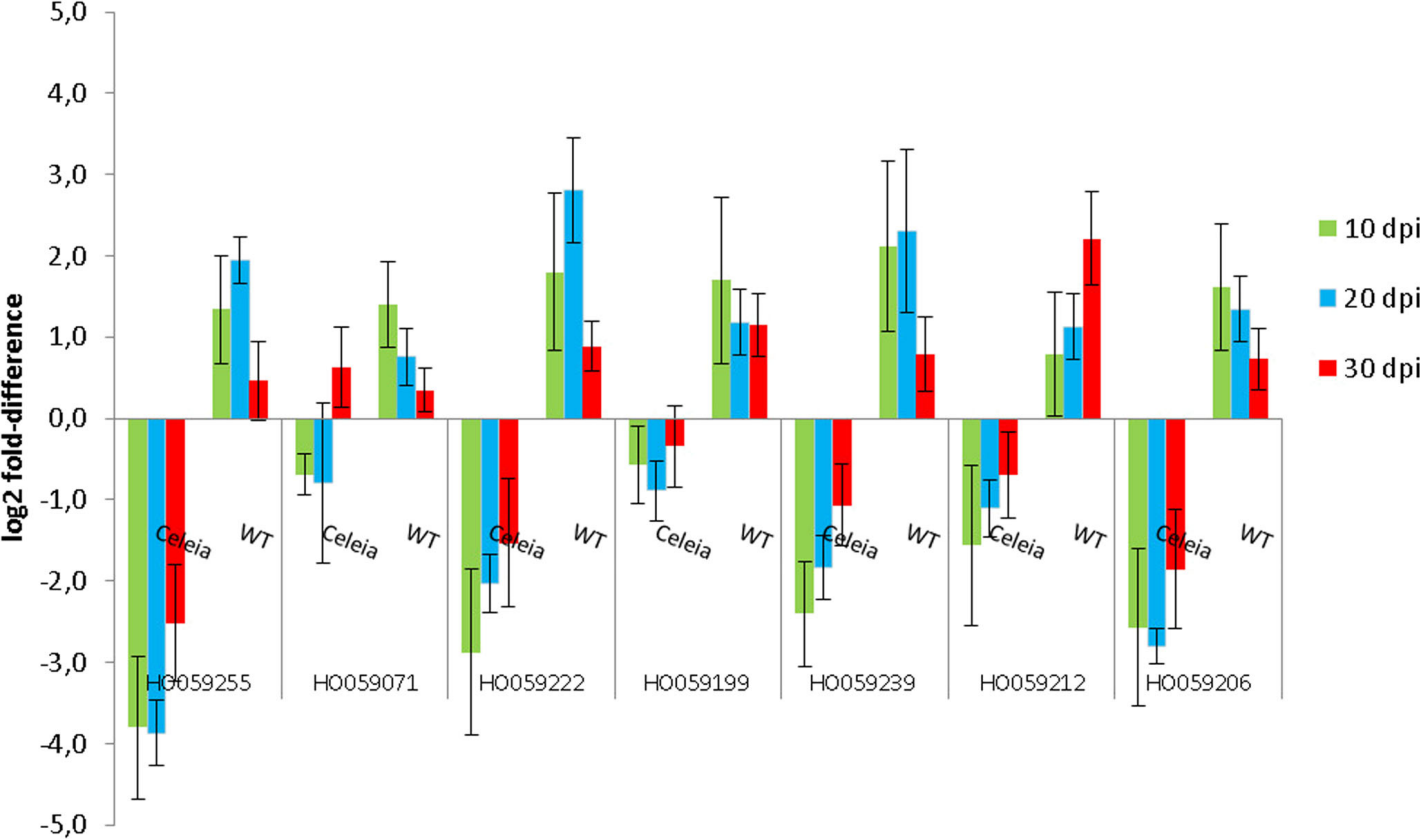
RT-qPCR relative expression levels of TDFs HO059255 (SKP1-like protein), HO059071 (Syntaxin), HO059222 (CDC48 protein), HO059199 (Puromycin-sensitive aminopeptidase), HO059239 (FK506 binding-like protein), HO059212 (Acyl-CoA-binding protein) and HO059206 (Furry protein) in susceptible Celeia and resistant Wye Target (*WT*) cultivars infected with *V. albo-atrum*. The values are relative to the level of expression in mock-inoculated plants at 10, 20 and 30 dpi. *Error bars* indicate SD of five biological replicates

TDF HO059255 is homologous to SKP1-like protein from *H. lupulus* L. available in GenBank. SKP1 protein is a component of the SKP1, cullin, F-box protein (SCF) complex, which regulates ubiquitination of proteins targeted for degradation by proteasome. Ubiquitination has been implicated in many cellular processes, including plant defence responses (Furlan et al. 2012), in which it is important in plant hormone signalling (Santner et al. 2009) and in the accumulation of nucleotide-binding leucine-rich repeat-type immune receptors (Cheng et al. 2011). Different components of the SCF complex have been monitored in pathogen-challenged plants for their expression patterns. Induced expression of SKP1 was observed in *N. benthamiana* after infection with potato virus X, as an indication of ubiquitination involvement in plant defence (Ye et al. 2013), and upregulation of Arabidopsis SKP1 (ASK1 and ASK2) was required for successful Agrobacterium-mediated plant transformation (Anand et al. 2012). We detected upregulation of SKP1 at 10 and 20 dpi in the incompatible interaction and strong downregulation in the compatible interaction (Fig. 5), which might imply involvement of ubiquitination in the incompatible interaction and lack of it in susceptible plants.

TDF HO059071 matched the syntaxin protein in grapes, with similarity to Arabidopsis syntaxin SYP22 (at5g46860), which is localized on the vacuolar membrane and the pre-vacuolar compartment and is involved in the vacuolar transport pathway (Uemura et al. 2010). SYP22 belongs to the family of SNARE (for soluble *N*-ethylmaleimide-sensitive factor protein attachment protein receptor) proteins, which are essential for vesicle fusion with the target membrane. Syntaxin proteins may play important roles in vesicle traffic associated with defence against fungal pathogens, as was shown for syntaxins of Arabidopsis PEN1 (SYP 121) and barley homolog ROR2, both involved in basal defence against powdery mildew (Collins et al. 2003; Bohlenius et al. 2010). We recorded increased expression of SYP22 homolog in resistant hop plants (Fig. 5) after inoculation with *V. albo-atrum,* suggesting elevated vacuolar transport and membrane fusion in the infected plants.

A similar expression pattern to SKP1 protein was found for TDF HO059222 (Fig. 5), which showed similarities to the *Medicago truncatula* sequence identified as a homolog of the Arabidopsis CDC48 protein (AtCDC48B), an AAA-ATPase that functions as a molecular chaperone in a wide variety of cellular activities. In yeast and human CDC48 homologs, VCP/p97, are characterized as ubiquitin-selective chaperones functioning in ubiquitin-proteasome-dependent protein degradation, autophagy, endosomal sorting and in chromatin-associated processes (Meyer et al. 2012). Induced expression of CDC48 was found in Arabidopsis on infection with TMV, whereby CDC48 interacts with the virus movement protein in the endoplasmic reticulum (ER) and controls virus movement by promoting degradation of the movement protein. The authors suggest a more general role of CDC48 in the maintenance of ER membranes under ER stress conditions (Niehl et al. 2012). It has also been shown that Arabidopsis CDC48 co-localizes in the ER and at the plasma membrane with somatic embrogenesis receptor-like kinase 1 (SERK1), a leucine-rich repeat receptor-like kinases, which is involved in a variety of signalling pathways (Aker et al. 2006), including Ve-mediated resistance in tomato challenged by *V. dahlia* (Fradin et al. 2011). Aker and de Vries (2008) further proposed a role of plant CDC48 as a chaperone for SERK receptors to promote correctly folded receptors in the ER and to remove misfolded receptors by the ubiquitin-proteasome degradation system. Moreover, the role of ER chaperones in Ve-mediated immunity in tomato has recently been reported (Liebrand et al. 2014). Upregulation of CDC48 in the hop-Verticillium incompatible interaction and downregulation in the compatible interaction (Fig. 5) implicate hop CDC48 in the immune reaction.

CDC48 also interacts with syntaxin and 14-3-3 protein, proteins involved in protein-protein interactions, which were also upregulated in our incompatible interaction, and it is a part of the ubiquitin-proteasome protein machinery as protein SKP1. The expression patterns of CDC48, its interaction with 14-3-3 and syntaxin, detection and expression pattern of SKP1, which is also part of the ubiquitin-proteasome protein machinery, suggest involvement of ubiquitination/vesicle trafficking in the hop-Verticillium immune reaction and lack of it in susceptible plants.

Highly upregulated expression (Fig. 5) in the incompatible interaction was also observed for TDF HO059199, with similarity to predicted puromycin-sensitive aminopeptidase in wild strawberry*.* Aminopeptidase have an important role in proteolytic events essential in a variety of physiologocal processes (Meinnel et al. 2006; van Endert 2011), and puromycin-sensitive aminopeptidase have been shown to be involved in degradation of polyglutamine sequences released by proteasomes (Bhutani et al. 2007), thus being part of the ubiquitin-proteasome protein machinery.

TDF HO059239, with similarity to FK506 binding-like protein, was highly expressed in the incompatible interaction and downregulated in the compatible interaction (Fig. 5). Plant FK506-binding proteins (FKBPs) have at least one specialized domain (FKBd), which acts in protein interactions and as an active site of peptidyl-prolyl isomerase. Through interactions with specific protein partners, FKBPs are implicated in cellular signalling, abiotic stress responses, photosynthesis and gene transcription, as reviewed by Gollan et al. (2012). Studies of the functional roles of this complex chaperone family are limited, and no involvement of FKBPs in biotic stress in plants has been well documented, but given the possibility that new interacting protein partners could extend their functions, we might speculate on the implication of hop FKBP in the resistant mechanism against Verticillium pathogen.

Another TDF with possible involvement in defence against microbial pathogens was found. HO059212 transcript was increasingly upregulated in resistant hops and downregulated in susceptible plants after infection by *V. albo-atrum* (Fig. 5). This transcript is a homolog to acyl-CoA-binding protein (ACBP) isolated from tea, *Camellia sinensis* (AEC10987), with 74 % identity to ACBP-6 Arabidopsis (at1g31812). ACBP-6 is one of six well-characterized ACBPs found in the Arabidopsis genome. They have various roles in plant lipid metabolism and are implicated in various biotic and abiotic stresses (Xiao and Chye 2009). A recent study showed that ACBP-6, ACBP-3 and ACBP-4, all cytosolic proteins, are all required for cuticle formation and defence against microbial and fungal pathogens, since the Arabidopsis mutants at these genes showed impaired cuticle development and affected resistant response (Xia et al. 2012). We detected an Arabidopsis ACBP-6 homolog as highly induced in the incompatible interaction, suggesting that acyl-CoA-binding proteins could be involved in the resistant response to infection by *V. albo-atrum*.

Upregulation in the incompatible interaction and downregulation in the compatible interaction was also observed for TDF HO059206 (Fig. 5), with similarity to a furry homolog-like protein from *Cucumis sativus*. This protein is conserved in eukaryotes from yeast to plants and mammals. In Drosophila, furry protein Fry is involved in controlling the morphogenesis of cell extensions such as bristles and epidermal hairs (Cong et al. 2001). Furry protein homolog Mor2 is required for establishing polarized cell growth in fission yeast (Hirata et al. 2002). The function of furry protein homologs in plants is still unknown, but they are likely to be involved in similar processes, e.g. controlling the morphogenesis of plant cell extensions, a process which might, due to the furry protein downregulation, be somehow inhibited in the hop-Verticillium compatible interaction.

## Conclusion

We present here a study of interactions between hop and *V. albo-atrum*, a vascular fungal pathogen threat to the hop industry, by monitoring the colonization pattern of fungus and by analysing expression patterns of fungal and hop TDFs selected out of differentially expressed TDFs isolated from inoculated susceptible and resistant cultivars in a time course experiment. Out of 92 TDFs obtained by cDNA-AFLP and ACP differential display, 84 TDFs showed similarity to plant proteins and 8 TDFs to fungal proteins. The discovered *in planta* expressed fungal genes might have some role in fungal virulence and should be further examined for their function, as well as plant genes with altered expression in compatible and incompatible interactions, to obtain a more complete picture of the resistance molecular mechanism. Two groups of TDFs, genes with altered expression and possible implication in immunity, were examined in this study by RT-qPCR. Induced expression in both interactions was shown for genes of PR proteins, aconitase, epoxy hydrolase, Cobra-like protein, MATE protein and 14-3-3 protein, while the group of genes upregulated in the incompatible and downregulated in the compatible interaction encode proteins involved in ubiquitination, vesicle trafficking, protein degradation and protein-protein interactions. The expression pattern of the last group of genes suggests involvement of ubiquitination/vesicle trafficking in the hop-Verticillium immune reaction and lack of it in susceptible plants. A common feature of the RT-qPCR expression patterns of the majority of the examined genes was that the highest values were reached at 20 dpi or values declined towards 30 dpi in the incompatible interaction. Similarly, fungal lectin gene showed its peak values at 20 dpi, whereas the peak of fungal biomass was at 15 dpi in the resistant cultivar, suggesting that the induced plant defence is strong enough to overcome fungus attack at these time points.

## Electronic supplementary material

Below is the link to the electronic supplementary material.

Supplemental Fig. S1 (DOCX 171 kb)

Supplemental Fig. S2 (DOCX 89 kb)

Supplemental Table. S1 (DOCX 17 kb)

Supplemental Table. S2 (XLSX 31 kb)

Supplemental Text. S1 (DOCX 1417 kb)

Supplemental Text. S2 (DOCX 2593 kb)
