# Supplementary material for: Different Gene Expressions of Resistant and Susceptible Hop Cultivars in Response to Infection with a Highly Aggressive Strain of Verticillium albo-atrum
Source: Plant Mol Biol Report. 2014 Aug 17;33(3):689–704. doi: 10.1007/s11105-014-0767-4 (PMC4432018; doi:10.1007/s11105-014-0767-4)

**Supplemental Fig. S1** Sequence distribution of annotated TDFs involved in a) biological processes, b) Molecular function, and c) Cellular components

a) Biological processes


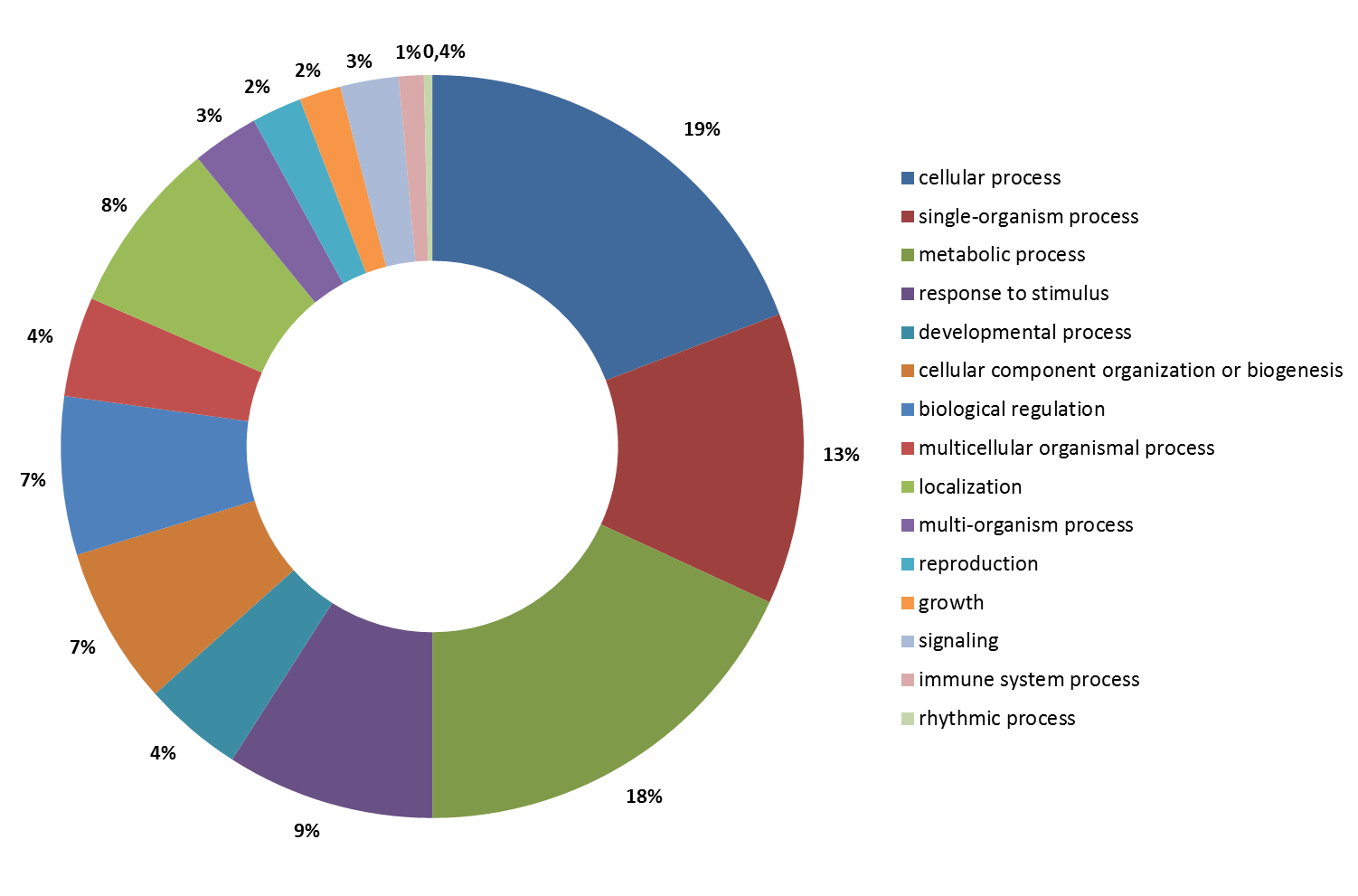


b) Molecular function


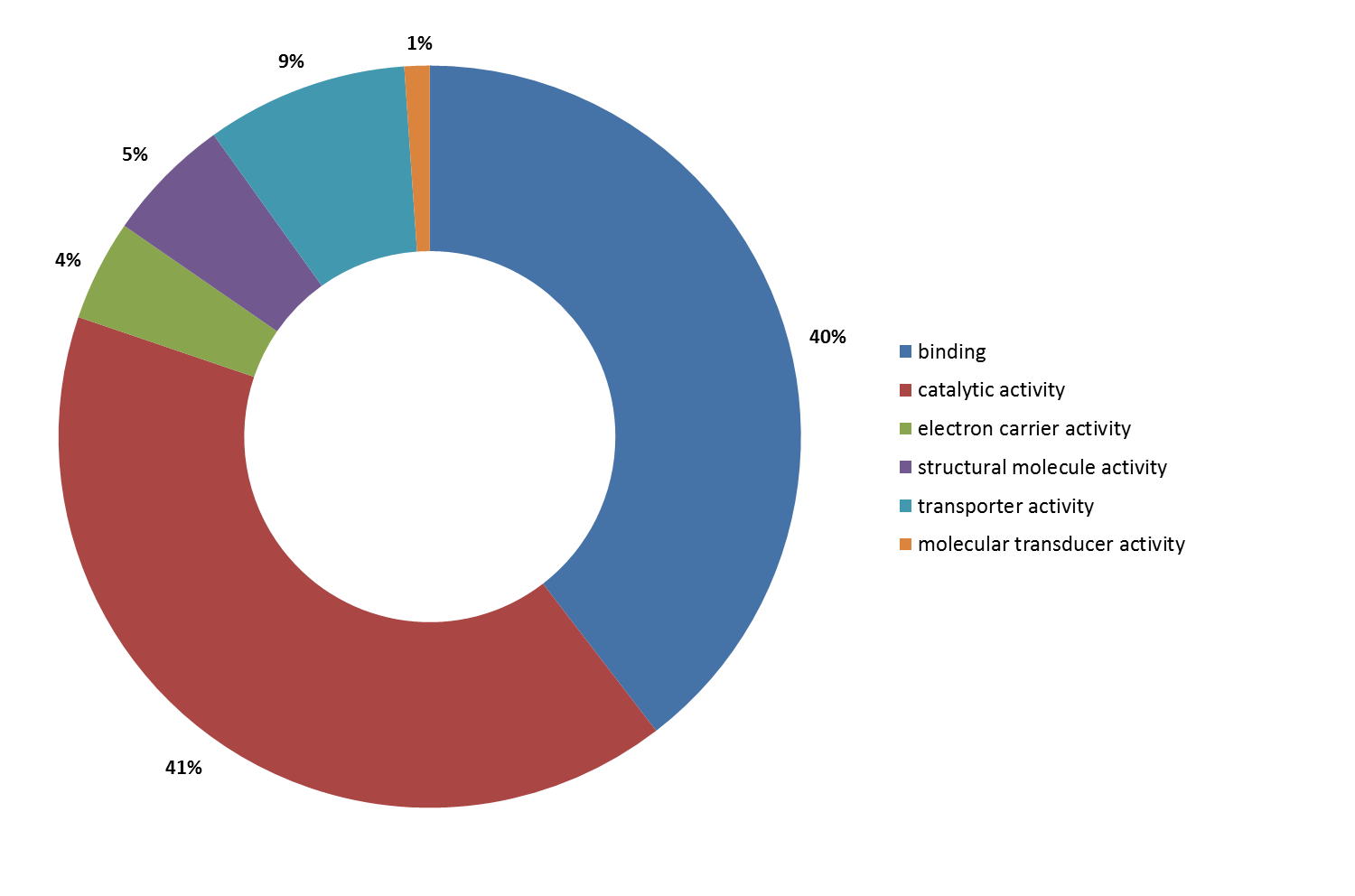


c) Cellular components


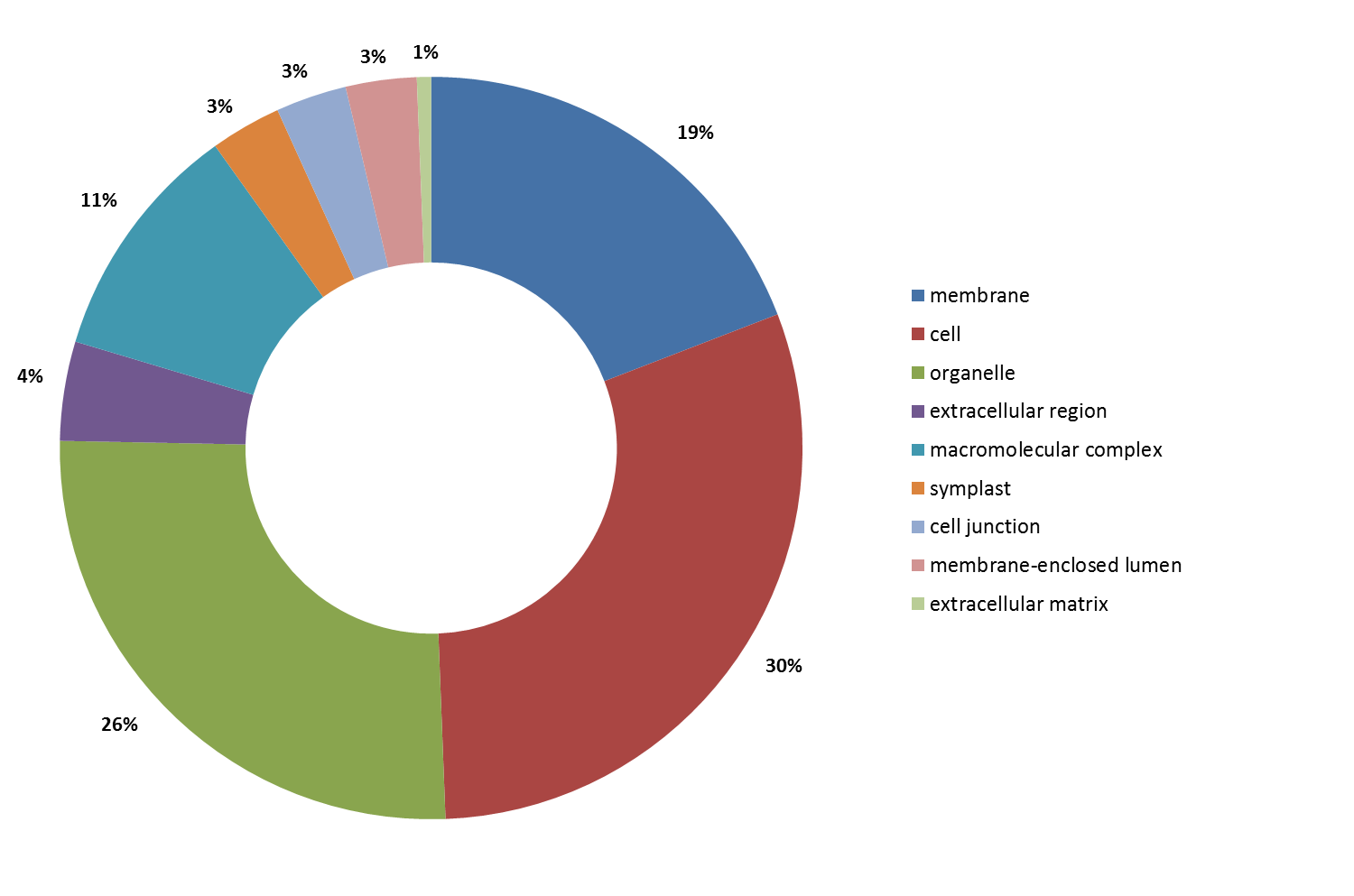

Supplement: Supplementary file 1 — (DOCX 171 kb) [file 11105_2014_767_MOESM1_ESM.docx]
