# Supplementary material for: Different Gene Expressions of Resistant and Susceptible Hop Cultivars in Response to Infection with a Highly Aggressive Strain of Verticillium albo-atrum
Source: Plant Mol Biol Report. 2014 Aug 17;33(3):689–704. doi: 10.1007/s11105-014-0767-4 (PMC4432018; doi:10.1007/s11105-014-0767-4)
Supplement: Supplementary file 2 — (DOCX 89 kb) [file 11105_2014_767_MOESM2_ESM.docx]

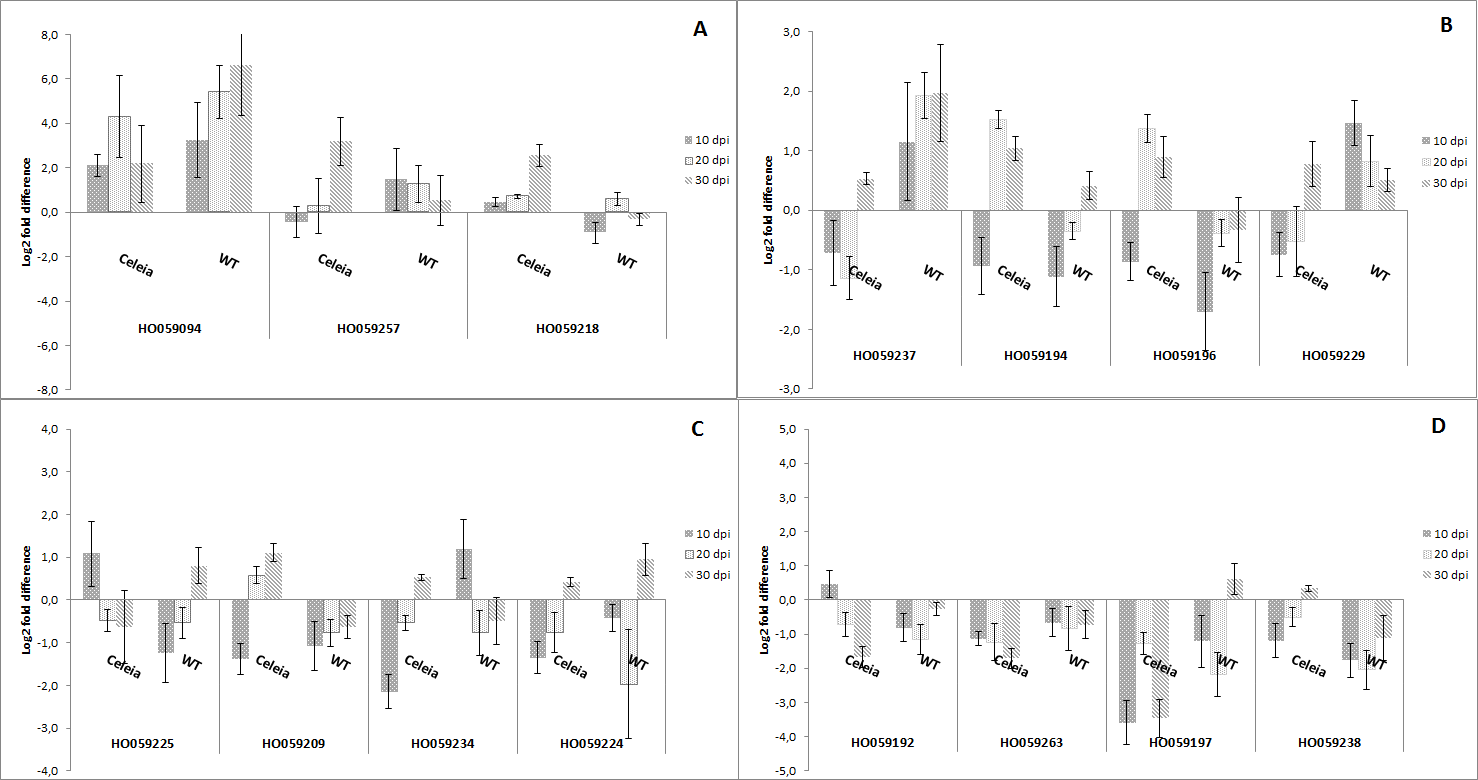


**Supplemental Fig. S2 Comparison of relative expression differences for analyzed genes: (A) HO059094, HO059257, HO059218; (B) HO059237, HO059194, HO059196, HO059229; (C) HO059225, HO059209, HO059234, HO059224; (D) HO059192; HO059263; HO059197; HO059238.** Relative expression ratios are given as the ratio of 'susceptible' compared to 'control' plants at 10, 20 and 30 dpi **for the susceptible ‘Celeia’ and resistant ‘Wye Target’ (WT) cultivars**. Error bars indicate SD of 5 biological replicates
