# Supplementary material for: Different Gene Expressions of Resistant and Susceptible Hop Cultivars in Response to Infection with a Highly Aggressive Strain of Verticillium albo-atrum
Source: Plant Mol Biol Report. 2014 Aug 17;33(3):689–704. doi: 10.1007/s11105-014-0767-4 (PMC4432018; doi:10.1007/s11105-014-0767-4)
Supplement: Supplementary file 3 — (DOCX 17 kb) [file 11105_2014_767_MOESM3_ESM.docx]

**Supplemental Table S1** Primer sequences used for Real-time amplification of 4 PR genes and 32 TDFs

| **Name** | **Forward primer 5’-3’** | **Reverse primer 5’-3’** |
| --- | --- | --- |
| PR1 | GAAGGTACCCTTATTGTTGTTGCA | GTTTGCGGGCACTACACTCA |
| PR2 | TCCAAACTCAGATCTCCAAAAGC | CTCACACTTGGCCAGAAATTCA |
| PR3 | TGTGCTGCAGCCAATTCG | CACTTGGTTTGCATTGGCTTT |
| PR5 | AAACCAATTCAACAACCGTGACT | CACCCTCCCGTAGTGGGACTA |
| HO059225 | ACGAAAAGGCGATGAGAGATG | GGCCTCGTTTACGGTGATGT |
| HO059222 | TGATGCCGACATACGCAAAT | CCCGACTCAGCAAACCTAAA |
| HO059237 | TCGAGAACACGCTGAAGCAA | TGCAATTCTACAGCCTAGGATCA |
| HO059238 | GTTCCTCCCCTTCGGAGTTG | TGCACCAAACGTCCAATAGTG |
| HO059239 | GCTCAGCCTGACAGCTACACT | AGGGCACTAGAGAAGACTTAGAATTT |
| HO059255 | GACGCAGATTTCGTCAAGGT | CAGGTCCAGCAAGCTCTTGAT |
| HO059259 | GGGCAAAAAAGGAGATAATATGGA | CTTGCAGCAATGTGGAATGTTC |
| HO059215 | GACACAGAGGTGGAGTTGGCTTA | TCCCTTATTCCTTACTGCTGATTAATC |
| HO059192 | GTGCAAGTGCCGTTCACATG | CGGTGCAGATCTTGGTGGTA |
| HO059193 | AGTCCAAGCCTAATTGGGTGAGT | AGCTGCTGAGAGCACTTTGTTG |
| HO059194 | GAGACCAGAACACCATAAGAGATTT | TGCCACTAGGGTTAGGGTTT |
| HO059196 | CAGATGCTTCGCAATTTCTG | GCCTCTTCCTCTGTCGCTACT |
| HO059199 | CTCACCGTTTGCTTCATTGGA | TGCTTCAAGGTTCTAGCAATGG |
| HO059197 | GAGCCAAGTGTTGCGACTCA | CGGAGTTCGAGAGGGTGTACA |
| HO059206 | TACTCCCTTCTCCTCCCTAAAGTACA | AGAGAAGTGGCTCTGCAGAACAC |
| HO059209 | GCCATGTAAAAAGTGGGAATTGA | CTGAGGTGAGGAGTTATGATTCGA |
| HO059212 | GCTGCTTCTTCCAGCAACTG | GGATGCATGGAAGGCTGTT |
| HO059218 | GAAGCCATGGTGAAGTATTCTAAA | GCTGTCTCCCTAGTATTCTTAAAGTG |
| HO059226 | TTGTACAGGGTTCGAACAAGTATTG | CTGCAGTCAAGTATACAGGTTTTCCT |
| HO059263 | ACCCTGCTGAAACCTTTGGT | TTCTAAATGATGTCAGAGCTTGAAT |
| HO059229 | ATCCTCAGGGTCAGGTGAGAAC | TGTTGATGATATTGGCCATGTTG |
| HO059234 | TTCACGCCGTTGAGCTTTTT | ACAACATCTCATCATCACAACGAA |
| HO059224 | TTGTGAGGGACCAACTTAGACAAC | GCAATGACGATGCTGACATTATCT |
| HO059232 | GAGGCCCTGCGTCTTCGT | CTGTTGTGCAAGTATAACCGTTAGTG |
| HO059244 | TAACTGGGAGCAAGAGGCAAA | GCATTGGCTAAGCTTCATGAGA |
| HO059257 | GAGTGTGAAACTCGAAGCCAATT | TATGCTAAGAGGCCGAGCTAGATT |
| HO059230 | GGAGTCTGGTTTTACTGGTCCTCTT | GTTGGAACTGTGATTTTTGATCCTT |
| HO059094 | TTGGAGTCCTGCAACTCTGGTA | CTGGTGACACCCTTGTGTTCA |
| HO059071 | AGATTAACACGGCGGTTTCG | TGCCTTGTCTTGTGCAGCTT |
| HO059113 | GGAAGAGTACAACAGAGGCAAG | CTTTGTTGCAGGACAACTTACA |
| HO059058 | GCCTTGTTGATTTTGACATTTGC | ATCTCCAAATAAGGTGATCCTCACA |
| HO059155 | GACACAGAGGTGGAGTTGGCTTA | TCCCTTATTCCTTACTGCTGATTAATC |
