# Supplementary material for: Different Gene Expressions of Resistant and Susceptible Hop Cultivars in Response to Infection with a Highly Aggressive Strain of Verticillium albo-atrum
Source: Plant Mol Biol Report. 2014 Aug 17;33(3):689–704. doi: 10.1007/s11105-014-0767-4 (PMC4432018; doi:10.1007/s11105-014-0767-4)
Supplement: Supplementary file 5 — (DOCX 1417 kb) [file 11105_2014_767_MOESM5_ESM.docx]

**Supplemental Text S1**

**Plant inoculation and symptom development**

Hop plants of Verticillium wilt susceptible cultivar Celeia (CEL) and resistant cultivar Wye Target (WT) were artificially inoculated by *V. albo-atrum* lethal pathotype PV1 by the root dipping method. Symptoms were assessed at three time points: 10, 20 and 30 dpi. The first symptoms, chlorotic and necrotic leaves, were observed in ‘Celeia’ at the 20 dpi stage at the level of 30% disease severity index, which progressed and became more severe at 30 dpi (53.8%). During the experiment, no visual symptoms were observed on resistant ‘Wye Target’ plants and mock-inoculated control plants.

**Fig S1** Symptom development, expressed as disease severity index (DSI) over time course post-inoculation. In the susceptible cultivar CEL, DSI continuously increased from 10-30 dpi, while resistant cultivar (WT) showed no visible symptoms


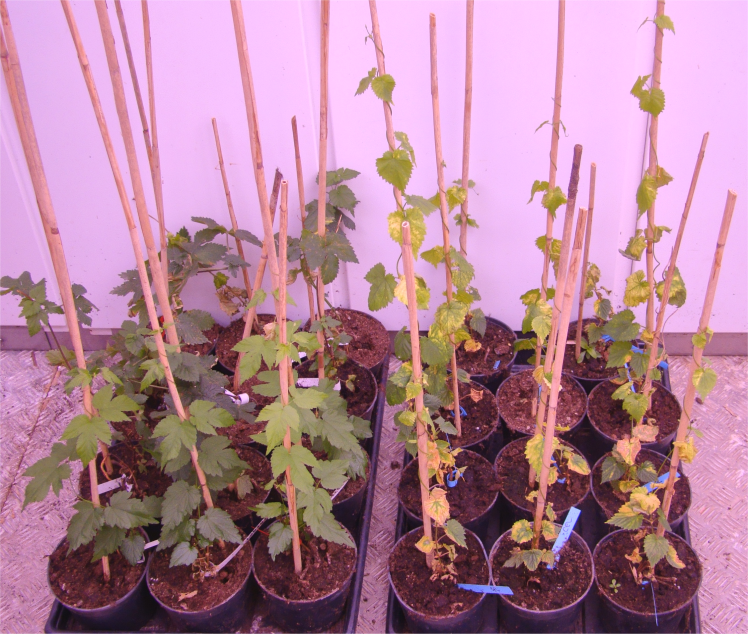


**Fig S2** Infected resistant WT (left) and susceptible CEL (right) plants at 30 dpi


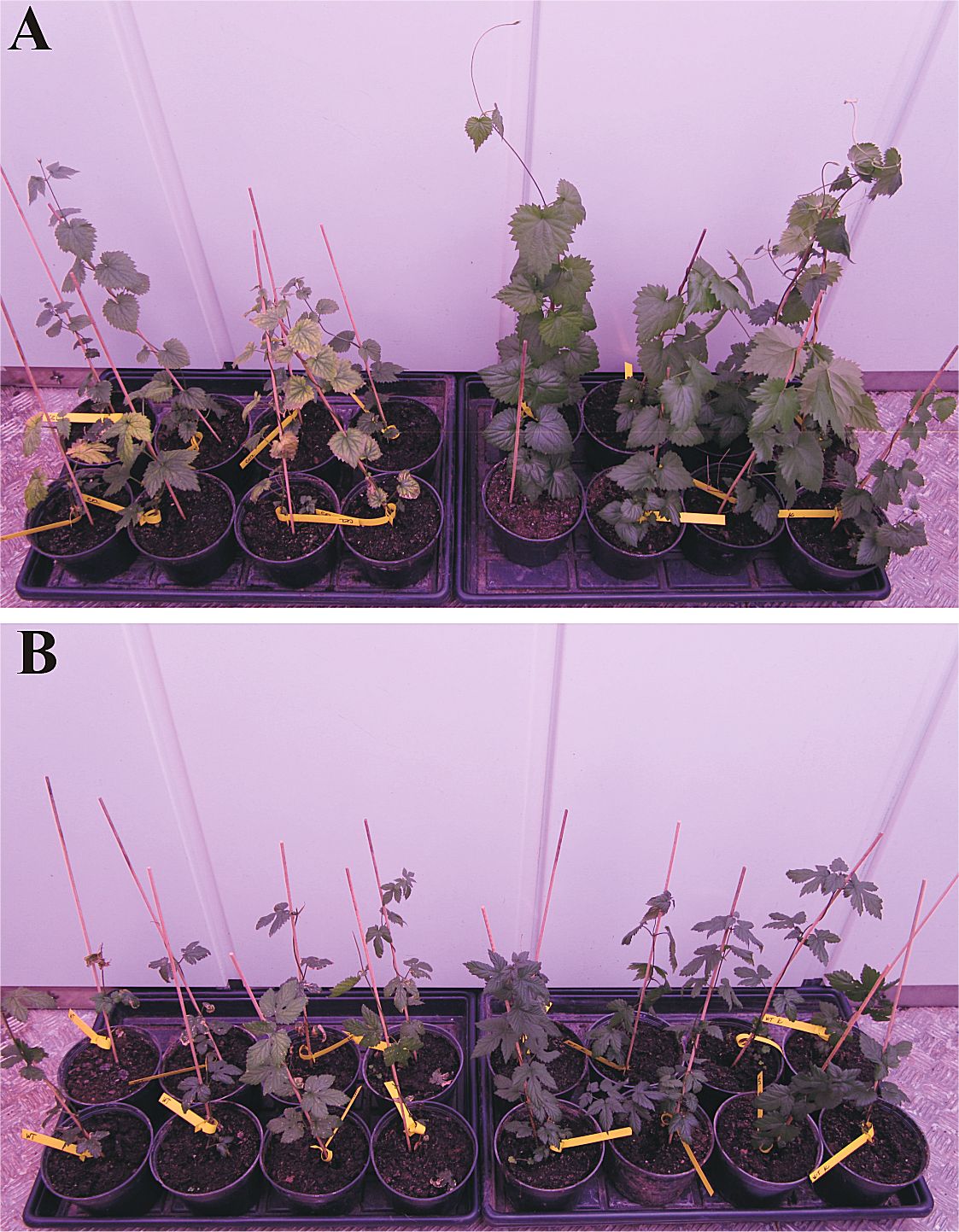


**Fig S3** Infected (left) and mock-inoculated Verticillium wilt susceptible hop cultivar Celeia (A) and resistant cultivar Wye Target (B) at 20 dpi
