# Supplementary material for: Different Gene Expressions of Resistant and Susceptible Hop Cultivars in Response to Infection with a Highly Aggressive Strain of Verticillium albo-atrum
Source: Plant Mol Biol Report. 2014 Aug 17;33(3):689–704. doi: 10.1007/s11105-014-0767-4 (PMC4432018; doi:10.1007/s11105-014-0767-4)
Supplement: Supplementary file 6 — (DOCX 2593 kb) [file 11105_2014_767_MOESM6_ESM.docx]

**Supplemental Text S2**

***Colonization of susceptible and resistant hop cultivars following infection by Verticillium albo-atrum***

**Introduction**

Verticillium wilt is a vascular disease of a broad range of plants, caused by the soil borne fungal species *Verticillium albo-atrum* and *V. dahliae.* In hop (*Humulus lupulus* L.), the disease caused by *V. dahliae* is comparatively rare and is mild in character, whereas *V. albo-atrum* shows a higher preference for hop and causes the majority of outbreaks in both mild and lethal forms (Neve, 1991). The appearance of disease forms is attributed to pathogen virulence, the sensitivity of hop cultivars and ecological factors (Isaac and Keyworth, 1948; Sewell and Wilson, 1984). In general, the mild form varies in intensity from year to year and rarely causes plant death, whereas lethal wilt is less influenced by seasonal climatic variations and causes very severe symptoms, with rapid plant withering.

The *V. albo-atrum* strain with increased virulence in hop (pathotype PV1) was first discovered in the UK in 1933 (Keyworth, 1942), followed by outbreaks in Slovenia in 1997 (Radišek et al., 2003) and Germany in 2005 (personal communication).

The disease cycle can be divided into dormant, parasitic and saprophytic phases. The fungus produces resting structures (melanized resting mycelium), which allow survival in the soil for up to 4 years (Sewell and Wilson, 1966). Following germination of resting structures in response to root exudates, hyphae invade the cortex directly without appressorium formation. Initial infections occur predominantly around the root tip or through wounds. Hyphae grow radially towards the stele and enter the xylem vessels after crossing the endodermal layer (Pegg and Brady, 2002). Colonization of xylem is accompanied by the production of spores, which travel up the plant with the transpiration stream (Sewell and Wilson, 1964). Spores are eventually trapped at trapping sites (vessel end walls or pits), where germination occurs in an attempt to penetrate the obstacle and colonize a new vessel. In this way, the fungus colonizes the entire plant and reaches the upper parts of the plant much faster than would be possible by mycelial growth alone. Heinz et. al (1998) found a cyclical alternation of pathogen colonization and host responses in tomato. The fungus emerges from xylem vessels only after tissue necrosis or abscission. Resting structures are formed during saprophytic colonization of dead tissues, thus providing an inoculum source for new infections.

Resistance to Verticillium wilt is a complex phenomenon, with both physiological and biochemical aspects. There is no immunity to infection and limited colonization can be observed even in the most resistant hosts (Pegg and Brady, 2002). Physiological responses are non-specific and associated mainly with physical restriction of fungal colonization and spread. Microscopic examinations of infected hop roots have revealed that epidermal and cortical cells respond to invading hyphae by callose and lignin deposition in cell walls and the formation of lignitubers. The main difference observed between susceptible and tolerant hop cultivars is strongly developed suberinization of the endodermal layer in the roots of tolerant plants (Talboys, 1958a). Although none of these responses can completely prevent vascular colonization, the amount of mycelium that reached the xylem vessels was shown to be significantly reduced in tolerant cultivars. Obstructions continue to form in xylem vessels in roots and stem, where extensive vessel blockage by tylosis was observed in tolerant cultivars (Talboys, 1985b). Vessel occlusion by gels and gums has been reported in many other host plants (Pegg and Brady, 2002). Resistant tomato plants are able to restrict fungal colonization to primary trapping sites by rapid coating of vessel walls with suberin (Gold and Robb, 1995).

Resistant responses of tolerant hop cultivars to infection by *V. albo-atrum*, as shown by early studies, were shown to be stronger suberinization of the endodermal layer in tolerant than in susceptible plants (Talboys, 1958a) and extensive vessel blockage by tylosis and xylem hyperplasia (Talboys, 1985b). We extended analysis of the colonization pattern in resistant and susceptible infected hop plants by histological examination in order to look for physical responses.

**Material and Methods**

*Sample preparation****:*** plants of resistant (‘Wye Target’) and susceptible (‘Celeia’) hop varieties were inoculated by the root dipping method using an inoculum (5x10^6^ conidia/ml) of *V. albo-atrum,* lethal pathotype PV1. Infected and mock-inoculated control plants were sampled at six time points: 3, 6, 10, 15, 20, 30 dpi.

*Histological examination****:*** fresh freehand sections were cut from the stems and roots of infected and non-infected plants with a razor blade at 3, 6, 10, 15, 20 and 30 dpi. The stem cuts were taken from the first internode (N1) above the collet (approx. 0.5 cm above the collet) and at about 5 cm above the collet (N2). The root cuts were made approximately 1 cm from the tip of the root (R). At least 10 cuts were taken for each sample. The cuts were transferred to AFA fixative (2.5 ml acetic acid, 6.5 ml formalin, 100 ml 50 % ethanol). Aniline Blue WS staining solution (10 g phenol, 10 ml glycerin, 10 ml lactic acid, 10 ml distilled water, 0.05 g aniline blue) was used for the staining procedure. The cuts were washed in distilled water for 10 min and transferred to the staining solution at 60 °C for 5 min. Stained cuts were washed in absolute ethanol for 1 min and mounted in Euparal mounting media on glass slides.

Morphological analysis was performed using an Olympus AX70 microscope with DP70 digital camera and cellSens digital imaging software (Olympus Corp.). Percentages of vessels with tyloses, hyphae and spores were estimated by counting the vessels in infected and control roots and two stem sections at each time point in five repetitions for each cultivar.

**Results and discussion**

Freehand sections from roots and stems of infected plants were examined under the microscope for physiological defence responses (Fig. S1). Synthesis of cell wall-coating materials such as callose, lignin or suberin around infected vessels was detected in the roots and stems of the susceptible cultivar (Fig. S1), while this response was less intensive in resistant plants. Fungal hyphae were found in around 15% of the vessels only in the susceptible cultivar (Fig. S2) in both roots and stems at later stages of infection (15, 20 and 30 dpi), although only a few vessels with spores were observed. Formation of tyloses in xylem vessels was intensive in the roots and stems of both cultivars (Fig. S3), with a higher percentage (up to 80%) of vessels obstructed with tyloses found in the stems of the susceptible cultivar (Fig. S4A, B). In roots, a different dynamic of tylose formation was observed between the two cultivars. In the roots of the resistant plants (Fig. S4B), the highest number of occluded vessels was found at 3 dpi (35%), with a decline towards 10 dpi (6%) followed by slight increase at 20 dpi (19%) and a final drop at 30 dpi (6%). In the roots of the susceptible cultivar (Fig. S4A), only a steady increase of vessels with tyloses over the time course was found, with the lowest number at 3 dpi (9%) and the highest at 30 dpi (65%). In two stems sections in both cultivars, tyloses occurred at all time points, reaching peaks at 80% and 67% in susceptible and resistant cultivars, respectively, at 20 dpi.

Non-specific plant responses to colonization in the form of physical obstructions were observed in the form of cell wall thickening, browning and tyloses, similar to that which was reported by Talboys (1958a,b). However, we did not observe stronger suberinization of the endodermis, extensive formation of tyloses and xylem hyperplasia in our resistant cultivar, Wye Target, as observed for the tolerant hop cultivar, Wye Challenger, in the Talboys study (1958 a,b). These differences can be attributed to the different hop cultivars studied, since the pedigree data of ‘Wye Challenger’ and ‘Wye Target’ reveal that these two hop cultivars contain different sources of Verticillium resistance known in hops (Neve, 1991), so different resistant responses would be expected. The formation of tyloses in the root xylem vessels in our resistant cultivar was the most interesting of the physical obstructions against spread of the fungus. At 3 dpi, when a small amount of fungus was present in the roots, the plant response was very strong, blocking around 35% of the vessels. Fungal colonization continued and reached a peak at 15 dpi while, at the same time, the number of vessels with tyloses decreased, being lowest at 10 dpi.


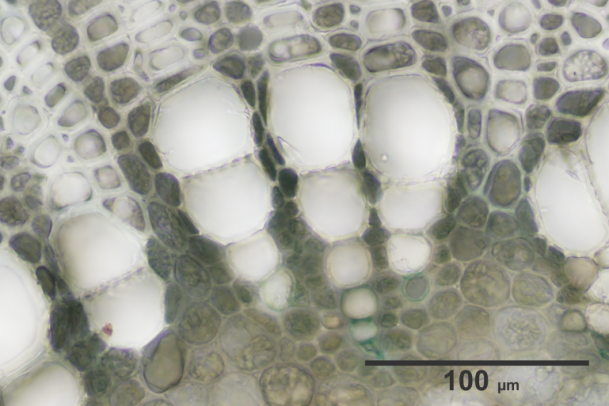

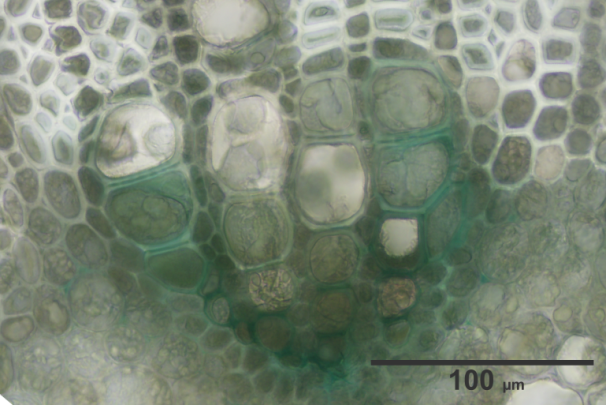


**Fig. S1** Cross sections of the stem of susceptible plants 15 dpi, stained with Aniline Blue WS. Xylem vessels of infected plants (right) showed strong cell wall deposition


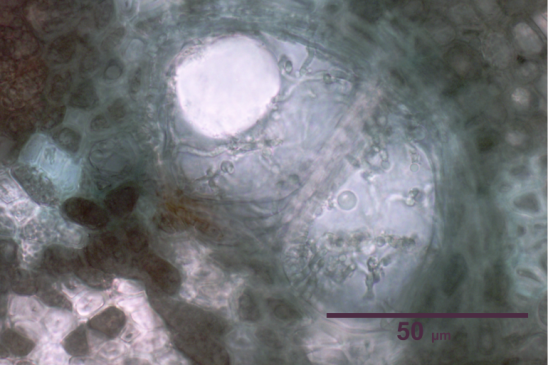

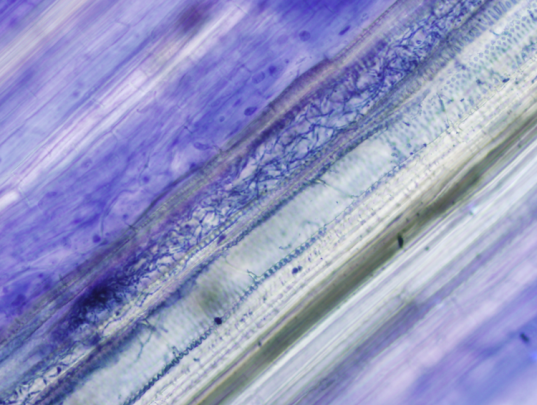


**Fig. S2** Cross-section (left, root) and longitudinal section (right, stem) of infected susceptible plants showing the presence of hyphae in xylem vessels, which appear in later periods post inoculation


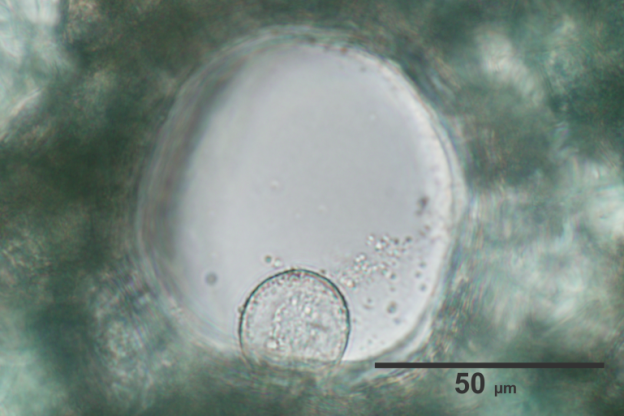

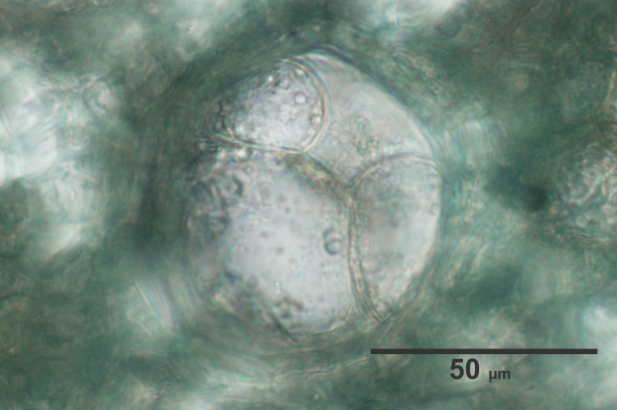


**Fig. S3** Formation of tyloses in root and stem xylem vessels was characteristic of both cultivars, as shown for the root cross-section of resistant plants at 10 dpi

**Fig. S4** Estimated percentages of xylem vessels containing tyloses in roots and two stem sections of susceptible (A) and resistant (B) hop cultivars over the time course (ND – not determined)

**References**

Gold J, Robb J (1995) The role of the coating response in Craigella tomatoes infected with *Verticillium dahliae*, race-1 and race-2. Physiol Mol Plant Pathol 47: 141-157. doi: 10.1006/pmpp.1995.1048

Heinz R, Lee SW, Saparno A, Nazar RN, Robb J (1998) Cyclical systemic colonization in *Verticillium*-infected tomato. Physiol Mol Plant Pathol 52: 385-396. doi: 10.1006/pmpp.1998.0163

Isaac I, Keyworth WG (1948) Verticillium wilt of the hop (Humulus lupulus). 3. A study of the pathogenicity of isolates from fluctuating and from progressive outbreaks. Ann Appl Biol 35: 243. doi: 10.1111/j.1744-7348.1948.tb07365.x

Keyworth WG (1942) Verticillium wilt of the hop (*Humulus lupulus*). Ann Appl Biol 29: 346-357. doi: 10.1111/j.1744-7348.1942.tb06138.x

Neve RA (1991) Hops. Chapman and Hall, London

Pegg GF, Brady BL (2002) Verticillium Wilts. CABI Publishing, Wallingford

Radišek S, Jakše J, Simončič A, Javornik B (2003) Characterization of *Verticillium albo-atrum* field isolates using pathogenicity data and AFLP analysis. Plant Dis 87: 633-638. doi: 10.1094/PDIS.2003.87.6.633

# Sewell GWF, Wilson JF (1964) Occurrence and dispersal of Verticillium conidia in xylem sap of the hop (*Humulus lupulus* L.). Nature 204: 901. doi: 10.1038/204901a0

# Sewell GWF, Wilson JF (1966) Verticillium wilt of the hop: the survival of V. albo-atrum in soil. Ann Appl Biol 58: 241-249. doi: 10.1111/j.1744-7348.1966.tb04383.x

Sewell GWF, Wilson JF (1984) The nature and distribution of *Verticillium albo-atrum* strains highly pathogenic to the hop. Plant Pathol 33: 39-51. doi: 10.1111/j.1365-3059.1984.tb00585.x

Talboys PW (1958a) Some mechanisms contributing to *Verticillium*-resistance in the hop root. T Brit Mycol Soc 41: 222-241. doi:10.1016/S0007-1536(58)80035-2

Talboys PW (1958b) Association of tylosis and hyperplasia of the xylem with vascular invasion of the hop by *Verticillium albo-atrum*. T Brit Mycol Soc 41: 249-260. doi:10.1016/S0007-1536(58)80037-6
